# The transcriptional program during germinal center reaction - a close view at GC B cells, Tfh cells and Tfr cells

**DOI:** 10.3389/fimmu.2023.1125503

**Published:** 2023-02-03

**Authors:** Annika C. Betzler, Alexey Ushmorov, Cornelia Brunner

**Affiliations:** ^1^ Department of Oto-Rhino-Laryngology, Ulm University Medical Center, Ulm, Germany; ^2^ Ulm University, Institute of Physiological Chemistry, Ulm, Germany

**Keywords:** germinal center (GC), GC B cells, Tfh cells, Tfr cells, transcriptional regulation, BOB.1/OBF.1, lymphoma

## Abstract

The germinal center (GC) reaction is a key process during an adaptive immune response to T cell specific antigens. GCs are specialized structures within secondary lymphoid organs, in which B cell proliferation, somatic hypermutation and antibody affinity maturation occur. As a result, high affinity antibody secreting plasma cells and memory B cells are generated. An effective GC response needs interaction between multiple cell types. Besides reticular cells and follicular dendritic cells, particularly B cells, T follicular helper (Tfh) cells as well as T follicular regulatory (Tfr) cells are a key player during the GC reaction. Whereas Tfh cells provide help to GC B cells in selection processes, Tfr cells, a specialized subset of regulatory T cells (Tregs), are able to suppress the GC reaction maintaining the balance between immune activation and tolerance. The formation and function of GCs is regulated by a complex network of signals and molecules at multiple levels. In this review, we highlight recent developments in GC biology by focusing on the transcriptional program regulating the GC reaction. This review focuses on the transcriptional co-activator BOB.1/OBF.1, whose important role for GC B, Tfh and Tfr cell differentiation became increasingly clear in recent years. Moreover, we outline how deregulation of the GC transcriptional program can drive lymphomagenesis.

## 1 Introduction

Germinal centers (GCs) are transient structures that form within B cell follicles of secondary lymphoid organs (SLOs), including lymph nodes (LNs), spleen and Peyer’s patches (PPs), in response to T cell dependent antigens (Ags) (1). Within GCs, B cells undergo intensive proliferation, immunoglobulin (Ig) gene somatic hypermutation (SHM), class-switch recombination (CSR) and affinity maturation. As a result, high affinity antibody (Ab) secreting plasma cells and memory B cells are generated (2). An effective GC response needs interaction between multiple cell types. These are in addition to follicular dendritic cells (FDCs), which are stromal cells residing in B cell follicles of SLOs, specifically B cells, T follicular helper (Tfh) cells and T follicular regulatory (Tfr) cells (2).

In LNs, B cells are organized in follicles within the outer cortex, while T cells are located in the surrounding paracortical area (3). Follicles are proximate to the subcapsular sinus (SCS), where Ags are delivered from the upstream lymphatic vessels (4). Ag presentation to B cells can take place in the SCS, the follicle or the paracortex (5). The spleen is composed of two compartments, the blood-filtering red pulp and the lymphoid white pulp (6). Similar to LNs, the white pulp is composed of B cell follicles and surrounding T cell areas, but follicles are additionally surrounded by the marginal zone. B cell follicles are separated by interfollicular regions in LNs and by arterial vessels in the spleen (4). GCs form within the center of the B cell follicles. Organization of the distinct areas and GC formation relies on the coordinated expression of chemokines and interaction of various cell types, as outlined in more detail in the following.

Naïve B cells located in the follicles of SLOs can be activated by binding Ags *via* their BCRs (4). Several mechanisms for B cell Ag encounter depending on factors like Ag size or their route of entry have been described (4, 5). Small Ags can diffuse through the SCS or follicular conduits to the follicle and activate B cells (4, 7). Larger Ags can be presented to B cells on the surface of DCs, FDCs or macrophages (7). The different mechanisms of Ag presentation to B cells are thoroughly reviewed elsewhere (4, 5, 7).

Upon BCR signaling, the BCR/Ag-complex is internalized and the Ag degraded to present its peptides on major histocompatibility complex (MHC)-class II to T cells (8). BCR engagement also increases expression of CCR7, which initiates B cell migration towards the B/T cell border, as its ligands CCL19 and CCL21 are expressed in the T cell zone by dendritic cells (DCs) and stromal cells (9). At the same time, naïve T cells located in the T cell zone recognize Ag presented by DCs. This initiates the differentiation towards a Tfh cell phenotype, which is characterized by the upregulation of CXCR5 and downregulation of CCR7 (10). This induces migration to the B/T cell border, where CXCL13, the ligand for CXCR5, is abundantly secreted by FDCs (11). Additionally, both B and T cells increase expression of Epstein-Barr virus-induced G protein coupled receptor 2 (EBI2), which directs their migration to the B/T cell border (12, 13). At this site, B cells present peptides of the Ag on MHC-II to CD4^+^ T helper cells thereby providing survival and co-stimulatory signals. Ag primed B and T cells form long-lived interactions, which results in full B cell activation (14). Subsequently, activated B and Tfh cells migrate into the center of the follicle, where B cells begin to proliferate and seed the GC. Therefore, B and T cells downregulate CCR7 and EBI2 while constantly expressing CXCR5, which enables them to move into the center of the follicle (15, 16). Moreover, B cells upregulate S1P receptor 2 (S1PR2), which promotes B cell migration to the follicle by binding S1P present in the follicle (17). S1PR2 also seems to be required for retention of Tfh cells in the GC (18). However, a subset of activated B cells does not enter the follicle, but migrate to extrafollicular regions in the spleen or in the medullary cords of LNs and differentiate into short-lived plasmablasts (19). Plasmablasts provide immediate help in recognition and elimination of the Ag by IgM secretion and subsequently, by IgM-mediated pathways (19, 20). Consequently, extrafollicular responses generate Abs faster, but with lower affinity compared to GC responses (19).

After migration into the follicle, the GC expands fast as a consequence of rapid proliferation and GC B cells push aside the resident follicular B cells (11). By day 7, the GC is fully established and polarized into dark and light zones. The dark zone is named for its densely packed appearance as rapidly proliferating B cells, termed centroblasts, reside in this area and undergo SHM (2). Centroblasts express CXCR5 and CXCR4 and are retained in the dark zone by the expression of CXCL12 released by local stromal cells (21). Over time, centroblasts reduce their cell division rates as well as their CXCR4 expression. These B cells are then termed centrocytes. Loss of CXCR4 expression allows their entry into the less densely packed light zone, attracted by the FDC released CXCL13, the ligand of CXCR5 (11).. In the light zone, affinity-based selection and CSR takes place. B cells compete for available Ag and T cell help. Those B cell clones which developed high affinity towards the Ag have a survival advantage over lower affinity B cell clones, which undergo apoptosis (2). GC B cells experience multiple rounds of re-entry into the dark zone before they finally differentiate into either plasma cells or memory B cells (11).

The formation and function of GCs is regulated by a complex network of signals and molecules at multiple levels. In this review, we highlight recent developments in GC biology focusing on the transcriptional program regulating the GC reaction. Besides B cells, we will also highlight the transcriptional program essential for Tfh and Tfr cell differentiation and function.

## 2 Transcriptional program regulating GC reaction

### 2.1 Transcriptional control of GC B cells

Several excellent reviews exist describing the transcriptional regulation of B cells during GC initiation, expansion and terminal differentiation into plasma or memory B cells in detail (1, 11, 22, 23). Therefore, we will just briefly highlight the main TFs regulating GC B cell differentiation and function, and focus on the transcriptional co-activator BOB.1/OBF.1, which was recently described to be essential for the GC transcriptional program.

#### 2.1.1 Master regulators of GC B cell expression programs

**Figure 1:**
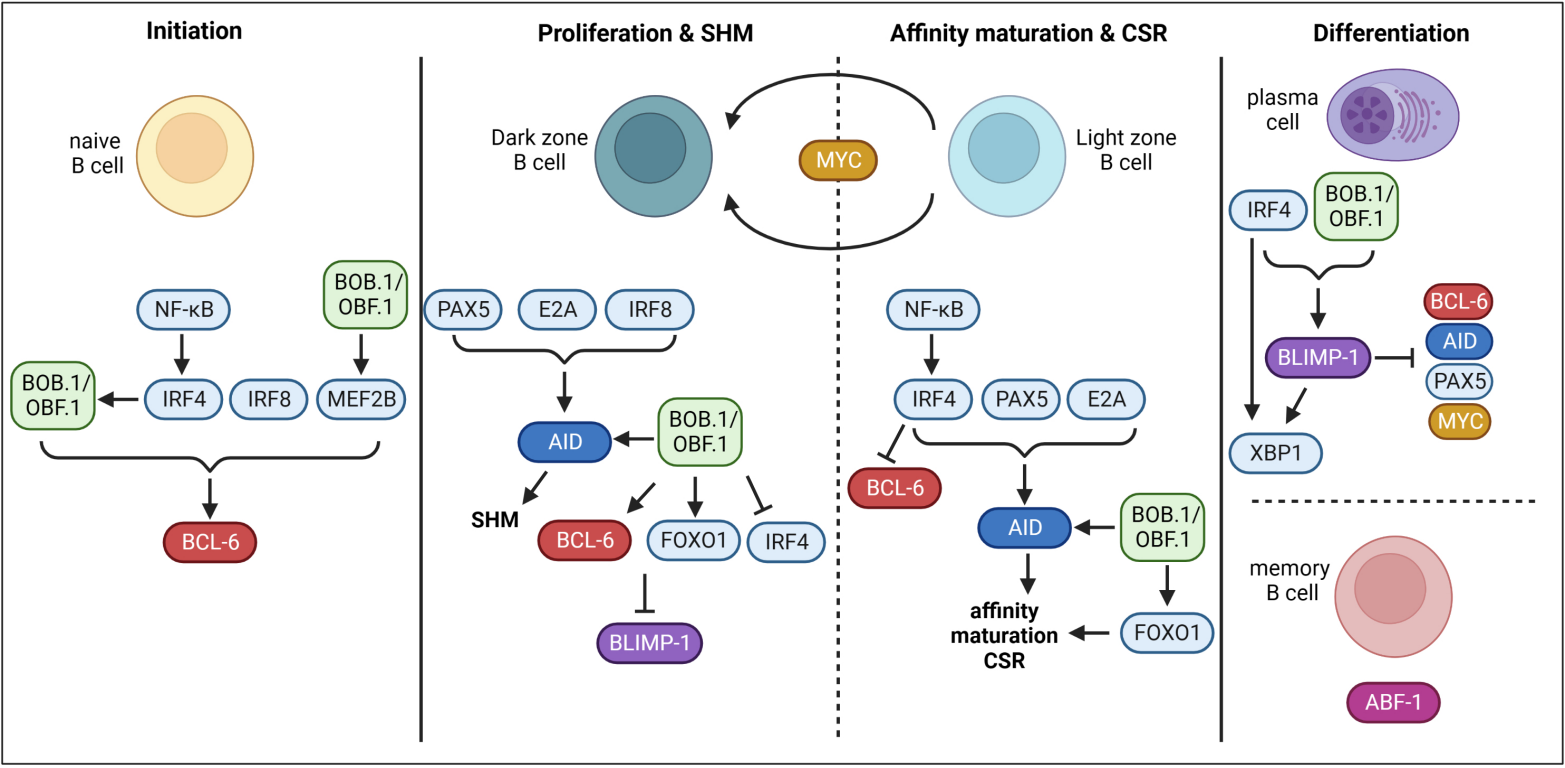
Transcriptional control of GC B cells. During GC initiation, TFs IRF4, IRF8 and MEF2B induce the expression of BCL-6. BOB.1/OBF.1 is also involved in the initiation of BCL-6 expression. Moreover, the transcriptional co-activator drives MEF2B expression. On the other hand, IRF4 seems to modulate the expression of BOB.1/OBF.1. In the dark zone, PAX5, E2A and IRF8 induce AID and therefore SHM. BOB.1/OBF.1 was also shown to be involved in AID, BCL-6 and FOXO1 induction. BCL-6 and FOXO1 inhibit BLIMP-1 and BOB.1/OBF.1 blocks IRF4 expression to prevent initiation of plasma cell differentiation at this stage. In the light zone, IRF4 is upregulated and prevents BCL-6 expression to terminate the dark zone program. IRF4, PAX5 and E2A promote AID expression and thereby affinity maturation and CSR. BOB.1/OBF.1 (POU2AF1) is again involved in regulation of AID and FOXO1 expression. The TF MYC probably regulates re-entry into the dark zone. IRF4 and BOB.1/OBF.1 are involved in driving BLIMP-1 expression. BLIMP-1 in turn represses BCL-6, AID, PAX5 and MYC and on the other hand facilitates expression of XBP1 together with IRF4 resulting in plasma cell differentiation. In contrast, ABF-1 induces memory B cell differentiation. This figure was created with BioRender.com.

Throughout the GC reaction, multiple regulatory and transcriptional networks characterized by the expression of ‘master TFs’ take place (**Figure 1**). BCL6 (B cell lymphoma 6 protein) is regarded as the master regulator of the GC reaction and essential for its initiation. The expression of BCL6 is induced by IRF4 (Interferon-regulatory factor 4), IRF8 (Interferon-regulatory factor 8) and MEF2B (Myocyte-specific enhancer factor 2B) (24–26). BCL6 acts as a transcriptional repressor on multiple levels during the GC reaction allowing the establishment of the GC B cell program. Firstly, BCL6 represses cell cycle regulators (*CDKN1A*/p21) (27) and genes of the DNA damage response pathway (*TP53*, *ATR*, *CHEK1*) (28–30), which permits rapid proliferation and SHM (31). Secondly, BCL6 regulates a transcriptional program facilitating B cell migration into the GC (32, 33). Thirdly, BCL6 represses genes necessary for B cell differentiation into premature plasma cells, thereby facilitating effective affinity maturation of GC B cells (34). In the dark zone, AID (Activation-induced cytidine deaminase), encoded by *Aicda*, is the key enzyme for SHM (35). Its expression is positively regulated by PAX5 (Paired box protein 5), E2A and IRF8 (25, 36, 37). Moreover, FOXO1 (Forkhead-Box-Protein O1) is highly expressed in the nucleus of GC dark zone B cells (38, 39). The activity of FOXO proteins is regulated by posttranslational modifications thereby regulating e.g. its shuttling between the cytoplasm and the nucleus, its stability or DNA binding (40). Phosphorylation of FOXO1 by the PI3K/AKT pathway induces its translocation from the nucleus to the cytoplasm resulting in its transcriptional inactivity (40, 41). On the other hand, phosphorylation at different sites by other protein kinases like RAS/mitogen-activated protein kinase promote nuclear localization and therefore the transcriptional activity of FOXO (41, 42). Several studies revealed that FOXO1 is essential for the GC dark zone program, as Foxo1-deficient GCs lacked a dark zone (38, 39, 43). Moreover, FOXO1 and BCL6 both repress BLIMP1 (B lymphocyte maturation protein 1), which is essential for plasma cell differentiation, thereby maintaining the GC dark zone program (38). In the light zone, affinity maturation and CSR take place. IRF4 is upregulated in light zone B cells, which in turn represses BCL6, thereby ending the dark zone transcriptome and establishing the light zone program (23). FOXO1 also seems to be involved in regulating light zone programs, as Foxo1-deficient GC B cells were described with impaired affinity maturation and CSR (38). CSR also relies on the expression of AID. During CSR, IRF4, FOXO1, PAX5, E2A and BATF (B Cell Activating Transcription Factor) are involved in AID regulation (38, 44–46). Upregulated IRF4 expression also induces BLIMP1 (encoded by *Prdm1*) expression (44, 47), which is essential for plasma cell development (48, 49). BLIMP1 further represses the expression of *Aicda*, *Bcl6* and *Pax5*, finally terminating the GC program (50, 51). BLIMP1 and IRF4 both activate XBP1 (X-box binding protein 1), which is required for Ab production and secretion (52–54). In contrast, ABF1 (Autonomously replicating sequence (ARS) binding factor 1) induces memory B cell differentiation and prevents plasma cell development (55). Another essential TF of the GC reaction is MYC. However, its role is incompletely understood. MYC is required during the very early phase of GC initiation, as its expression is induced immediately after immunization (56, 57). However, in dark zone B cells its expression is repressed by BCL-6. Later, MYC becomes re-expressed in a subset of light zone B cells and probably regulates their re-entry into the dark zone (56, 57).

#### 2.1.2 BOB.1/OBF.1 controls the GC B cell transcriptional program

Another factor essential for the GC reaction is BOB.1/OBF.1 (B cell Oct binding factor 1/Oct-binding factor 1), also known as OCA-B (octamer coactivator from B cells). BOB.1/OBF.1 is encoded by the *Pou2af1* (POU domain class 2-associating factor 1) gene. BOB.1/OBF.1 is expressed in both B and T lymphocytes and acts as transcriptional co-activator of octamer-dependent transcription (58). The octamer motif is found in regulatory elements of B and T cell specific genes (59–61). BOB.1/OBF.1 interacts with TF of the Oct-family (Oct-1 and Oct-2) and enhances their binding selectivity to the octamer motif (5’-ATGCAAAT-3’) and their transcriptional activity (62–65). Thus, the ternary complex built by BOB.1/OBF.1 together with Oct-1 or Oct-2 on the DNA octamer motif regulates expression of genes essential for lymphocyte physiology (62, 65–70). Analysis of BOB.1/OBF.1-deficient mice revealed several defects in early and late B cell antigen-independent maturation. BOB.1/OBF.1 is critical for early B cell development in the bone marrow, as BOB.1/OBF.1-deficient mice feature a developmental block at the pro/pre B cell stage (71, 72). Consequently, the number of peripheral B cells is severely reduced in these mice (73). Additionally, BOB.1/OBF.1 is essential for the maturation of follicular B cells, since BOB.1/OBF.1-deficient mice show increased levels of immature but decreased numbers of mature B cells (71, 73). Besides, the number and function of marginal zone B cells is affected in the absence of BOB.1/OBF.1 (74). However, the most striking characteristics of BOB.1/OBF.1-deficient mice are the complete absence of GCs and consequently dramatically reduced levels of class-switched Igs (71–73, 75). However, it was completely unsolved at which stage of B cell development BOB.1/OBF.1 expression is essential for GC formation. By establishing a mouse system allowing the conditional deletion of BOB.1/OBF.1 at different time points of B-cell maturation, we have recently shown that an efficient GC reaction needs BOB.1/OBF.1 expression during the complete B-cell ontogeny (76). Notably, in Pou2af1^fl/fl^ x Cγ1-Cre mice, in which BOB.1/OBF.1 is specifically deleted in GC B cells four days after immunization also lack GCs (76). This finding proves that this deficiency is a GC B-cell autonomous defect and not exclusively a consequence of defective early B-cell maturation. The TF IRF4 seems to regulate the induction of BOB.1/OBF.1 expression during the early GC reaction initiation (24). For a long time, GC-specific BOB.1/OBF.1 target genes were largely unknown. In 1998, NF-κB, Oct-2 and BOB.1/OBF.1 were shown to cooperatively regulate the promoter activity of the chemokine receptor Cxcr5 in B cells (77). Thus, reduced Cxcr5 expression observed in conventional BOB.1/OBF.1-deficient mice might contribute to the absence of GCs in these animals as a consequence of impaired B lymphocyte migration (69, 77). In addition, BOB.1/OBF.1-deficient B cells of postnatal day 9 showed a dramatic reduction in the frequency of migration towards Cxcl13 indicating an impaired Cxcr5 function in these cells (78). These findings in combination with observed diminished FDC numbers and reduced Cxcl13 expression in the absence of BOB.1/OBF.1 suggest that reduced lymphocyte numbers in SLOs of these mice could be a consequence of defective Cxcl13-Cxcr5 signaling (78). In 2006 Spi-B, which is essential for GC formation and maintenance (79), was revealed as BOB.1/OBF.1 target (80). Ten years later, a ternary complex on the *Bcl6* promoter formed by BOB.1/OBF.1 and Oct-TFs was described, activating *Bcl6* transcription and thereby promoting Tfh cell development (69). In 2020, it was demonstrated that BOB.1/OBF.1 also regulates BCL-6 expression in GC B cells (81). The authors found that BOB.1/OBF.1 forms a ternary complex with Oct-2 and the TF MEF2B, which occupies and regulates a locus control region that regulates BCL-6 expression (81). Most recently, combining multiple experimental approaches a map of direct BOB.1/OBF.1 target genes was defined. Precisely, transgenic mice expressing tagged versions of Oct-1, Oct-2 and BOB.1/OBF.1 were generated, which allowed for ChIP-sequencing and the identification of their DNA-binding sites (68). This strategy in combination with the analysis of specific histone marks to examine chromatin activity status as well as RNA-sequencing experiments allowed the identification of several BOB.1/OBF.1 target genes (68). These data show that BOB.1/OBF.1 and Oct-TFs regulate the GC transcriptional program by binding to multiple genes encoding TFs essential for GCs including *Bcl6*, *Myc*, *Foxo1*, *Mef2b, Aicda* and *Spi1*in primary B cells (68) (**Figure 1**). Moreover, the authors provide evidence that BOB.1/OBF.1 is also required for the maintenance of the GC transcriptional program. In the absence of BOB.1/OBF.1 the GC transcriptional program is disrupted as genes essential for GC maintenance (*Bcl6*) are downregulated and genes initiating the plasma cell program are upregulated (I*rf4*) (68). Together, these results highlight BOB.1/OBF.1 as a key player in maintaining the GC transcriptional program by activating BCL-6 and repressing IRF4. When the expression of BOB.1/OBF.1 is abolished, the BCL-6 pathway is diminished and IRF4 expression gets enhanced, which results in the termination of the GC program and the initiation of plasma cell differentiation (68). In addition, BOB.1/OBF.1 seems to be essential for the induction of *Prdm1*, thereby contributing to plasma cell differentiation (70).

### 2.2 Transcriptional control of GC T cells

#### 2.2.1 Transcriptional regulation of Tfh cell differentiation

**Figure 2:**
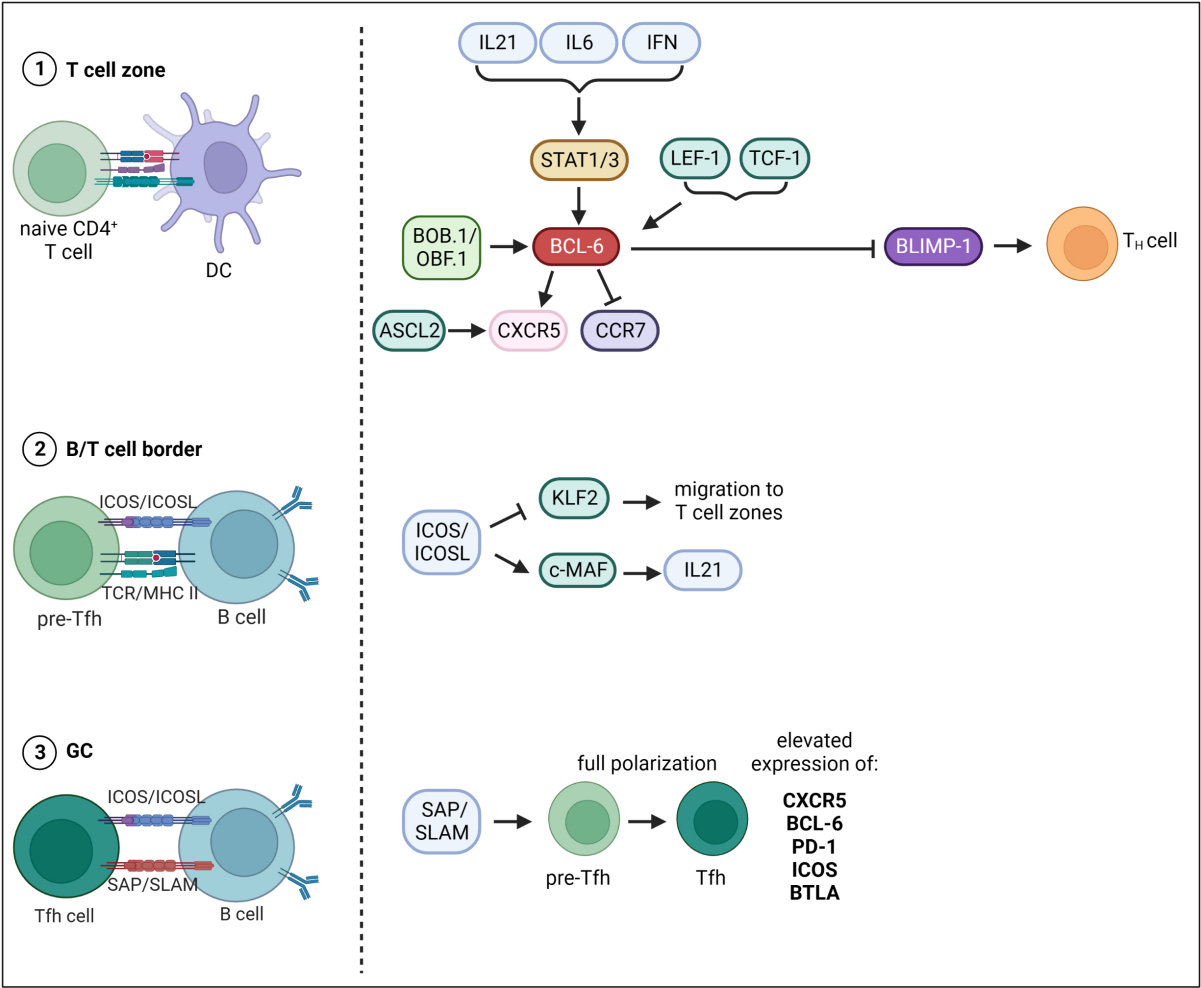
Transcriptional control of Tfh cell differentiation. Tfh cell differentiation occurs in three main steps. The first step of Tfh cell differentiation is the interaction of naïve CD4^+^ T cells with DCs in the T cell zone. The expression of BCL-6, master TF of Tfh cells, is induced on multiple levels. Cytokines including IL-21, IL-6 and type I IFNs (IFN-a/b) drive Bcl-6 expression via the activation of STAT1/3. Besides, TFs LEF-1 and TCF-1 as well as the transcriptional co-activator BOB.1/OBF.1 together with TFs Oct-1/Oct-2 induce Bcl- 6 expression. BCL-6 in turn represses Blimp-1 and TFs favoring development into TH subpopulations. BCL-6 and the TF ASCL2 induce the expression of CXCR5 and suppresses CCR7 expression inducing migration of pre-Tfh cells to the B/T cell border. The second step of Tfh cell differentiation then occurs upon interaction of pre-Tfh cells with Ag-specific B cells in the B/T cell border. ICOS/ICOSL signaling represses the TF KLF2 thereby inhibiting migration back to the T cell zones. ICOS signaling also induces the TF c-MAF, which facilitates IL21 expression. The third step of differentiation occurs within GCs, where SLAM/SAP binding is required to stabilize B/T cell interactions. Here, pre-Tfh cells differentiate into Tfh cells, a further polarized state characterized by highest expression levels of BCL-6, CXCR5, PD-1, ICOS and BTLA. This figure was created with BioRender.com.

The differentiation of Tfh cells is a multistage process, whereby multiple TFs that either promote or repress Tfh cell fate are involved (**Figure 2**). The first step of Tfh cell differentiation is the interaction of naïve CD4^+^ T cells with DCs in the T cell zone. CD4^+^ T cells undergo cell fate decision either towards T helper (T_H_) cell subsets or Tfh cells. There is evidence that Tfh cell development is initiated already during DC priming after viral infection (82). Thereby, ICOS (Inducible T-cell COStimulator) seems to be required to induce Bcl6 expression (82). Another study also suggested a direct link between TCR signal strength, IL-2 production and Tfh cell fate decision (83). Indeed, CD4^+^ T cell commitment towards Tfh cell fate was reported within first rounds of cell division only two days after viral infection (82). Cells either start to express Bcl6, the master regulator of Tfh cell differentiation, or its antagonist Blimp-1, which favors T_H_ cell development. The expression of BCL-6 is induced on multiple levels. Several cytokines including IL-21, IL-6 and type I IFNs (IFN-α/β) induce *Bcl6* expression *via* the activation of STAT1/3 (84–87). The TFs LEF-1 (Lymphoid enhancer binding factor 1) and TCF-1 (T cell factor-1) promote *Bcl6* expression by targeting signaling molecules upstream of BCL-6 like IL-6R and ICOS (88). TCF-1 was also shown to directly bind to the *Bcl6* promoter facilitating its expression (89). BCL-6 in turn represses Blimp-1 and TFs like T-bet, Gata3 and RORγt, which favor the development into T_H_ subpopulations (90). BCL-6 and also the TF ASCL2 (Achaete-Scute Family BHLH Transcription Factor 2) induce the expression of CXCR5 and suppresses CCR7 expression inducing migration of pre-Tfh cells to the B/T cell border (91, 92). The second step of Tfh cell differentiation occurs upon interaction of pre-Tfh cells with Ag-specific B cells in the B/T cell border. B/T cell interaction functions as feedback loop as it further drives both Tfh as well as GC-B cell development. The B/T crosstalk happens in a peptide/MHC II and ICOS/ICOSL dependent manner. ICOS/ICOSL signaling represses the TF KLF2 (Krüppel-like Factor 2), which is required to maintain the Tfh cell phenotype as KLF2 promotes the expression of genes that mediate the migration to the T cell zones and away from the follicles (93). ICOS signaling also induces the TF c-MAF, which directly binds to the *Il-21* promoter inducing the expression of this key-Tfh cytokine (94). Within GCs, SLAM/SAP binding is required to stabilize B/T cell interactions (95). Here, pre-Tfh cells differentiate into Tfh cells, a further polarized state characterized by highest expression levels of BCL-6, CXCR5, PD-1, ICOS and BTLA (96). What happens to Tfh cells after they have fully differentiated and provided help for GC B cells is very well understood. It is supposed that Tfh cells can exit a GC and (i) migrate to another GC, (ii) transitionally reside in an adjacent follicle and re-enter the same follicle again or (iii) completely exit the GC and acquire a memory-like phenotype (97, 98).

#### 2.2.2 Requirement of BOB.1/OBF.1 for Tfh cell differentiation

The transcriptional co-activator BOB.1/OBF.1 plays a central role in controlling the transcriptional program of GC B cells as outlined above. BOB.1/OBF.1 has long been considered a B cell specific factor, but in recent years a contribution of BOB.1/OBF.1 expression in T cells to the GC reaction became evident. Several recent studies revealed a high BOB.1/OBF.1 expression in murine and human Tfh cells (69, 99–101) and BOB.1/OBF.1 ablation results in reduced Tfh cell numbers in mice (69, 102–105). Most recently, a patient with mutation in the *Pou2af1* gene resulting in the absence of BOB.1/OBF.1 protein was reported who also featured reduced Tfh cell numbers (103). Moreover, BOB.1/OBF.1 deficiency prevented the differentiation of CD4^+^ T cells into Tfh cells in an autoimmune mouse model (106). In line, BOB.1/OBF.1-deficient mice revealed a significantly reduced Tfh cell compartment in response to viral infection (102). However, when mice were reconstituted with BOB.1/OBF.1-deficient T and WT B cells prior to influenza infection Tfh cells developed normally (102). In contrast, mice reconstituted with BOB.1/OBF.1-deficient B and WT T cells revealed impaired Tfh cell differentiation, which initially led to the conclusion that this defect might be B-cell mediated (102). Nonetheless, Stauss et al. reported a prominent reduction of Tfh cells in BOB.1/OBF.1-deficient mice upon SRBC immunization and revealed a CD4^+^ T cell autonomous defect by performing reconstitution experiments (69). In addition, loss of BOB.1/OBF.1 was found to be associated with reduced Bcl-6 expression in Tfh cells from mixed bone marrow chimeras. ChIP experiments of BOB-deficient CD4^+^ T cells maintained under Tfh inducing conditions revealed binding of BOB.1/OBF.1 together with Oct-1 and Oct-2 to an octamer motif within the *Bcl6* promoter (69). In the same year, the study by Yamashita et al. contradicted these findings (99). They reported increased Tfh percentages in BOB.1/OBF.1-deficient animals suggesting a BOB.1/OBF.1 related mechanism that limits TCR-mediated Tfh cell expansion (99). Different experimental settings regarding immunization and mouse strains might account for this discrepancy. Yamashita et al. only analyzed PD1^+^ CXCR5^+^ Tfh cells of spleens by flow cytometry and their further experimental approach was predominantly *in vitro* (99). Stauss et al., reported a reduction of multiple Tfh cell subsets (PD1^+^, ICOS^+^ BTLA^+^) in spleen, LNs and PPs of BOB.1/OBF.1-deficient mice by flow cytometry and immunofluorescence (69). Moreover, they showed that BOB.1/OBF.1 transactivates *Bcl6* and *Btla* promoters (69). In 2022, Lombard-Vadnais et al., could show that Tfh differentiation is blocked at the early Tfh maturation stage when BOB.1/OBF.1 is absent in hematopoietic cells (104). However, in line with the observation of Karnowski et al. (102), the authors here also suggested that BOB.1/OBF.1 plays a T cell extrinsic role in Tfh cell differentiation and that instead its expression in B cells promotes Tfh cell maturation (104). In the same year, a study by us revealed reduced Tfh cell numbers when BOB.1/OBF.1 was deleted tissue-specific in a CD4- or IL21-Cre dependent manner (105). In particular, the reduction in Pou2af1^fl/fl^ x IL21-Cre mice, which deletes BOB.1/OBF.1 predominantly in Tfh cells, provides evidence that Tfh cell development and function requires T-cell specific BOB.1/OBF.1 expression. Since IL21 is also required for Tfh cell differentiation (84), crossing Pou2af1^fl/fl^ mice to IL21-Cre mice results in BOB.1/OBF.1 deficiency already in pre-Tfh cells. Thus, BOB.1/OBF.1 might play a role already in pre-Tfh cells and their subsequent differentiation. Moreover, T and Tfh cell-specific BOB.1/OBF.1 deletion also resulted in impaired GC formation (105). Thus, BOB.1/OBF.1 expression in T cells is also required for efficient GC formation. Consequently, the contribution of BOB.1/OBF.1 to the GC reaction cannot be exclusively attributed to its expression in B cells. The fact that the reduction of GCs was more prominent in Pou2af1^fl/fl^ x CD4-Cre mice suggests that BOB.1/OBF.1 expression in T cells is required early during the process of GC initiation. This is in line with findings of *Pou2af1* being highly expressed in early stage GC-Tfh cells (88). Altogether, the role of BOB.1/OBF.1 for Tfh cell differentiation was controversially discussed in the past years due to different experimental approaches. However, tissue-specific deletion of BOB.1/OBF.1 emphasizes the Tfh cell intrinsic role of BOB.1/OBF.1 for Tfh cell maturation and function (105). Yet, since signals from GC B cells are required for Tfh maintenance, a combination of both the B cell and Tfh cell intrinsic role of BOB.1/OBF.1 might contribute to both efficient GC B and Tfh cell development.

#### 2.2.3 Transcriptional control of Tfr cells

Tfr cells are a subpopulation of regulatory T cells (Tregs) and are found within GCs in mice and humans (107–112). Their assumed function is the suppression of the GC reaction by repressing excessive Tfh and GC B cell proliferation thereby promoting selection of high affinity B cell clones (107–109, 113). Tfr cells share characteristics of both Tregs and Tfh cells and it is supposed that Tfr cells have different phenotypes at different stages of maturation or in various locations (111, 114, 115). Tfr cells express FoxP3 (Forkhead box P3), CTLA-4 (Cytotoxic T-lymphocyte-associated Protein 4), GITR (Glucocorticoid-induced TNFR-related gene) and BLIMP-1 similar to Tregs (108, 111, 116, 117). On the other hand, they express BCL-6, CXCR5, ICOS, PD1 and SAP typical for Tfh cells (108, 111, 117). It is assumed that the majority of Tfr cells arises from natural Tregs (107, 108). However, there is evidence that they can also derive from naïve FoxP3-negative CD4^+^ T cells (118).

**Figure 3:**
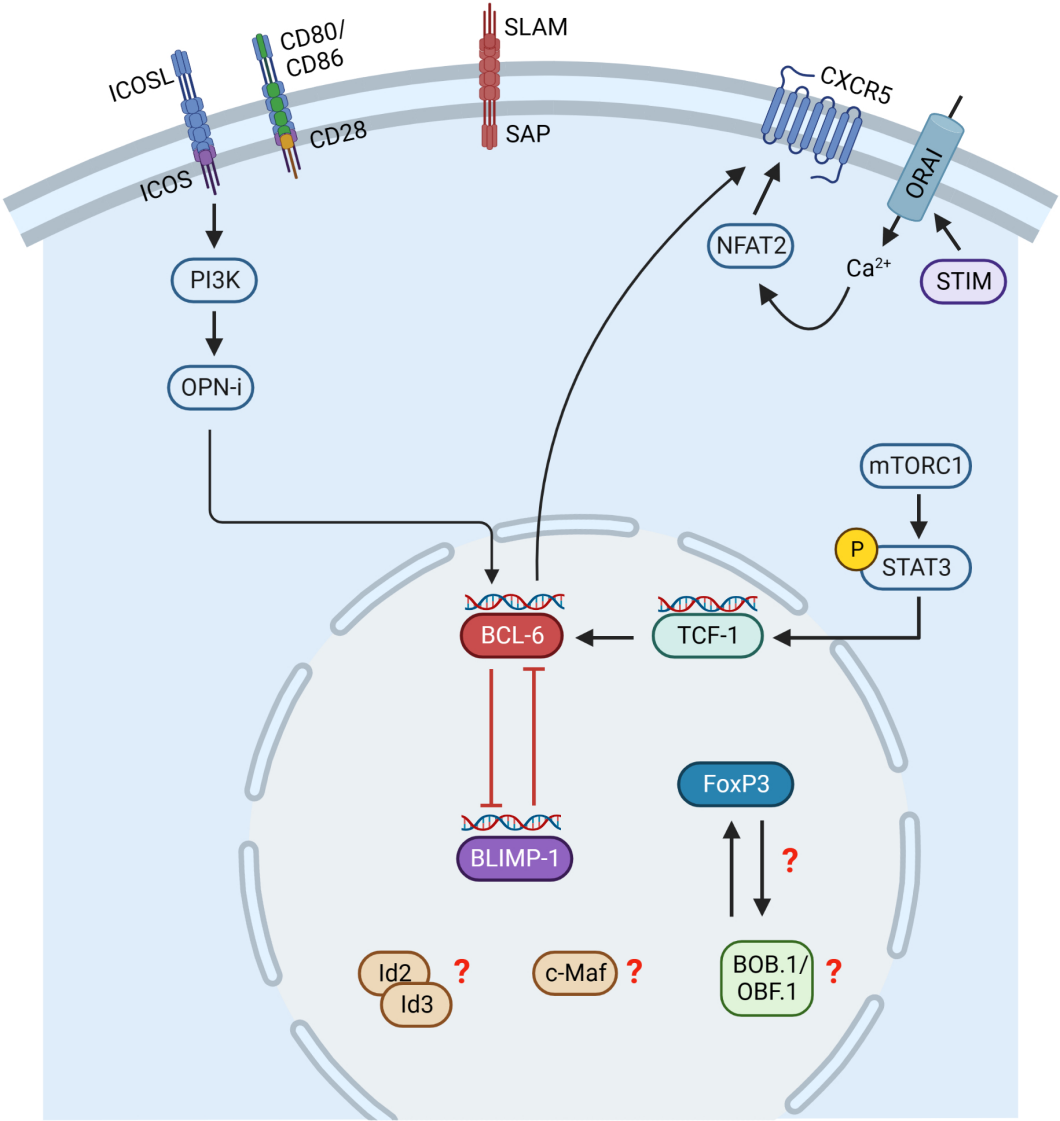
Transcriptional control of Tfr cell differentiation. Initial Tfr cell differentiation depends on CD28 and ICOS signaling, whereas interaction with B cells via SLAM/SAP is required for their full differentiation. Tfr cells express both BCL-6 and BLIMP-1 and possibly their balanced regulation is essential for Tfr cell differentiation. BCL-6 is regulated by ICOS and mTORC1. ICOS induces PI3K and its subunit p85α interacts with osteopontin (OPN-i). OPN-i translocates to the nucleus preventing BCL-6 from ubiquitin-dependent proteasome degradation. mTORC1 induces phosphorylation of STAT3, activating TCF-1 expression, which upregulates BCL-6. Stromal interacting molecules (STIM) and ORAI proteins mediate Ca2+ entry inducing NFAT2, which upregulates CXCR5. Id2/Id3, c-Maf and BOB.1/OBF.1 are also involved in Tfr cell differentiation, but the exact mechanisms are incompletely understood so far. This figure was created with BioRender.com.

Similar to Tfh cells, the Tfr cell differentiation process is initiated by Ag presentation by DCs (**Figure 3**). However, it is not completely solved which Ag signals initiate Tfr cell differentiation since Tfr cells respond to both foreign and self-Ags, but more strongly to self-Ags (118, 119). Besides Ag-receptor stimulation, Tfr cell differentiation depends on co-stimulation through CD28 and ICOS (108, 120). However, Tfr and Tfh cells were shown to have different TCR repertoires and that of Tfr cells resembles more that of Tregs (121). This allows the conclusion that Tfr cells develop in a polyclonal and Ag-independent manner from Tregs (113). Initial Tfr cell differentiation relies on CD28 and ICOS signaling, whereas interaction with B cells *via* SLAM/SAP is required for their full differentiation (90, 108, 120). Besides Tfh cells, BCL-6 is also the key TF regulating Tfr cell differentiation. In contrast to Tfh cells, Tfr cells express both BCL-6 and BLIMP-1 (108). It is assumed that the balanced regulation by these two opposing TFs is essential for efficient Tfr differentiation and function, since BCL-6 ablation results in the absence of Tfr cells (108), while lack of Blimp-1 increases Tfr cell numbers, however, with reduced suppressive capacity (122). Therefore, it is hypothesized that BCL-6 is essential for Tfr generation, whereas BLIMP-1 is required to limit and control Tfr cell numbers (122). Another key TF of Tfr cells is FoxP3, which is the known master regulator of Treg cells. FoxP3 modifies the Tfh transcriptional program to induce a Tfr-like functional state (123) as well as to maintain the Tfr cell transcriptional program (123). Thus, FoxP3 is required for Tfr cell identity and suppressive function (123). GC homing in Tfr cells is initiated by the TF NFAT2 (Nuclear factor of activated T cells 2), which upregulates CXCR5 expression, thus playing a comparable role to ASCL2 in Tfh cells (124). In this context, store-operated Ca^2+^ entry (SOCE) through Ca^2+^ release-activated Ca^2+^ (CRAC) channels mediated by stromal interacting molecules (STIM) and ORAI proteins were shown to promote Tfr cell differentiation through NFAT-mediated IRF4, BATF, and BCL-6 TFs (125). Subsequently, CXCR5 expression is maintained by BCL-6 (107, 108). BCL-6 in turn is regulated by ICOS and mTORC1 in Tfr cells. ICOS induces PI3 kinase and its subunit p85α interacts with osteopontin (OPN-i) (126). OPN-i translocates to the nucleus preventing BCL-6 from ubiquitin-dependent proteasome degradation (126). Additionally, mTORC1 was shown to promote Tfr cell differentiation *via* STAT3/TCF-1/BCL-6 pathway (127). TFs Id2/Id3 (Inhibitor of DNA Binding 2/3) are also involved in Tfr cell differentiation. Id2 and Id3 expression decreases upon TCR stimulation allowing the induction of the Tfr cell specific transcriptional program including CXCR5 and IL10 expression (128). The sclerostin domain-containing protein 1 (SOSTDC1) was shown to block the WNT-β-catenin axis thereby facilitating Tfr cell differentiation, possibly by upregulating FoxP3 and CXCR5 (129). In addition, the ablation of c-Maf, which is also involved in Tfh cell differentiation, resulted in reduced Tfr cell numbers (130). However, the exact mechanisms by which c-Maf regulates Tfr cell differentiation remain to be elucidated.

#### 2.2.4 Role of BOB.1/OBF.1 in regulatory T cells

As BOB.1/OBF.1 was previously shown to be required for Tfh cell differentiation by regulating BCL-6 expression, as outlined in the previous chapter, it is likely that BOB.1/OBF.1 might also be involved in Tfr cell differentiation. BOB.1/OBF.1 was already linked to FoxP3 and Tregs, since an altered expression of *Pou2af1* in Tregs was revealed (111, 131, 132). In detail, *POU2AF1* was shown to be up-regulated in circulating human blood Tfr cells compared to Tfh and Treg cells (111). Additionally, *Pou2af1* expression is suppressed in stimulated FoxP3^+^ cells in comparison to FoxP3^-^ cells and FoxP3 occupies the *Pou2af1* promoter (131). On the other hand, co-culture experiments of T cells with Tregs resulted in an upregulation of *Pou2af1* expression in suppressed compared to non-suppressed T cells (133). Metenou et al. reported a downregulation of *Pou2af1* in Tregs from patients with chronic infection compared to healthy individuals (132). B cells co-cultured with Tfr cells downregulate *Pou2af1* expression (134), which further suggests a relation between Tfr cells and *Pou2af1* expression in a broader context. Ablation of BOB.1/OBF.1 in T cells using Pou2af1^fl/fl^ x CD4-Cre mice resulted in an increase of Treg cells in our study (105). Additionally, these mice revealed an impaired Ag-specific immune response (105). These results imply that BOB.1/OBF.1 expression in both Tfh and Tfr cells might balance affinity maturation. Indeed, the exact role of Tfr cells for affinity maturation is not clear. Originally, Tfr cells were thought to control the production of Ag-specific Abs, while more recent studies suggest Tfr cells to restrain affinity maturation (15). However, the exact role of BOB.1/OBF.1 in Tregs and Tfr cells especially in terms of affinity maturation needs to be further elucidated. So far, the role of BOB.1/OBF.1 for Treg/Tfr cell development and function and whether its expression in these cell subsets promotes or restrains affinity maturation is not clear. RNA-seq analysis of BOB.1/OBF.1-sufficient compared to BOB.1/OBF.1-deficient CD4^+^ T cells of immunized mice revealed the histone methyltransferase Kmt2d (Lysine Methyltransferase 2d) among the top differentially expressed genes. We found increased *Kmt2d* expression in BOB.1/OBF.1-deficient CD4^+^ T cells compared to WT CD4^+^ T cells (105). Kmt2d deficiency was reported to compromise Treg cell development (135), possibly due to its requirement for the induction of FoxP3 expression (135). Mechanistically, Kmt2d catalyzed H3K4 methylation at distant enhancers *via* chromatin looping thereby contributing to the induction of FoxP3 expression (135).

Altogether, only little is known about the function of BOB.1/OBF.1 in Tregs so far. There is evidence that BOB.1/OBF.1 has a role in Tregs and/or Tfr cells, as several studies described altered *Pou2af1* expression in these cells in different experimental settings, as outlined above. However, there is no definite knowledge about the role of BOB.1/OBF.1 in these cells at the current time. Some studies report an induction of *Pou2af1* expression in Tregs or Tfr cells, while others describe a suppression. Further studies are required to get deeper insights into how BOB.1/OBF.1 expression is regulated, particularly in Tfr cells, as well as how Treg-specific BOB1.OBF.1 contributes to the balanced regulation of induction and suppression of an ongoing GC reaction.

## 3 Deregulation of the GC transcriptional program in disease

### 3.1 TFs hijacked during tumorigenesis of GC-derived lymphomas

The GC reaction relies on the interplay of multiple TFs regulating distinct phases of GC formation, as described in the preceding chapters. These TFs can be hijacked to drive tumorigenesis of GC-derived lymphomas. The majority of non-Hodgkin lymphomas (NHLs) derive from GC B cells including diffuse large B cell lymphoma (DLBCL), follicular lymphoma (FL) and Burkitt lymphoma (BL) (136, 137). The exact signaling pathways and mechanisms driving B cell lymphomagenesis are thoroughly reviewed elsewhere (136–140). In this chapter, we highlight the contribution of key TFs of the GC reaction as well as of the transcriptional co-activator BOB.1/OBF.1 to malignant transformation.

#### 3.1.1 MYC

The gene encoding the TF MYC was the first to be linked to GC B cell lymphomagenesis even before its role in GC physiology was resolved (141, 142). MYC is expressed during the early initiation phase of the GC and subsequently repressed by BCL-6. However, it becomes re-expressed in a subset of light zone B cells later on (56, 57). The ectopic and constitutive expression of MYC is a frequent feature of GC-derived lymphomas. Chromosomal translocations of *MYC* are found in all BLs and in a proportion of DLBCLs (143, 144). In most cases, the *MYC* gene translocates into the Ig heavy chain locus and is brought under the transcriptional control of the Ig enhancer elements resulting in its transcriptional deregulation (141–143). Moreover, it is thought that translocations remove BCL-6 binding sites on the MYC promoter (56). Consequently, repression of MYC by BCL-6 is prevented, leading to ectopic MYC expression. A further link between MYC and cancer is that functions of the TF include controlling proliferation, cell growth, apoptosis and DNA replication (136, 145). Aberrant MYC expression thereby drives tumorigenesis.

#### 3.1.2 BCL-6

As BCL-6 is a key TF for GC reaction, its dysregulation is also involved in the pathogenesis of GC-derived lymphomas. Aberrant BCL-6 expression is triggered by different mechanisms. Translocations or mutations of BCL-6 are found in DLBCL and FL (146). The most frequent cause of altered BCL-6 expression is translocation. These translocations replace the regulatory region of *BCL-6* with promoters from Ig and non-Ig genes, preventing downregulation of BCL-6 (147–149). In addition, GC-derived lymphomas display various somatic mutations in the BCL-6 regulatory region that occur separately from translocations, avoiding the auto-regulatory mechanisms of BCL-6 to repress its own expression or block binding of suppressive IRF4 (150–152). BCL-6 dysregulation is also caused by indirect mechanisms including mutations of *MEF2B*. These mutations are commonly found in DLBCL and FL. MEF2B transactivates BCL-6. Thus, *MEF2B* mutations contribute to lymphomagenesis by deregulating BCL-6 expression (26, 153).

#### 3.1.3 FOXO1

FOXO1 is a well-known tumor suppressor, but recent studies also reported tumor-promoting functions of the TF for different tumor entities (154–157). In GCs, FOXO1 instructs the dark zone program (38, 39). Recurrent somatic mutations of FOXO1 are common in B-NHL of GC origin including GCB-DLBCL, FL and BL (157–161), but their pathogenetic significance might differ in these entities. In GCB-DLBCL, the FOXO1 mutations are associated with poor prognosis and they are more frequent in relapsed or primary refractory cases (159, 160, 162), whereas in BL, there is no correlation with outcome or progression of the disease (161). The mechanisms regulated by the FOXO1 mutations are also obscure. Importantly, both activating (repressing FOXO1 inactivating phosphorylation) and inactivating, (targeting DNA-binding domains) are common in GCB-DLBCL (163). Physiologically, PI3K/AKT phosphorylates FOXO1 leading to its nuclear export and transcriptional inactivation (162, 164). Consequently, it has been hypothesized that in BL and GCB-DLBCL, mutations in the N-terminal hotspot help FOXO1 to escape the AKT-mediated inactivation (157). Nevertheless, following studies in BL cell lines and original tumors demonstrated strong nucleal FOXO1 expression virtually in all cases independently on the mutational status (161, 165). Moreover, the postulation on high AKT activity in BL has been questioned (166). Overall, there is a lack of correlation between PI3K/AKT signaling status, FOXO1 mutations, and subcellular localization, suggesting that selection of FOXO1 mutations during lymphomagenesis cannot be explained solely by the effects of PI3K/AKT on FOXO1.

Although the mechanisms underlying the oncogenic effects of activating and inactivating FOXO1 mutations are still obscure, the essential role of FOXO1 in maintenance of BL is clearly documented (165). A recent study provides evidence that FOXO1 mutations encode for proteins that mimic signaling and transcriptional features of positively selected B cells (167). Consequently, expansion of FOXO1 mutant B cells is favoured (167).

#### 3.1.4 *PRDM1/*BLIMP-1

Disrupted *PRDM1* gene expression is frequently found in Activated B-cell-like (ABC) DLBCLs, which is a post-GC malignancy. However, it is supposed that the disease is caused by a blockade of the terminal differentiation at the plasmablast stage (136, 138). This blockade seems to be due to BLIMP-1 inactivation, caused by truncations, mutations or frameshift deletions of the *PRDM1* gene (168–170). As *PRDM1* is directly repressed by BCL-6, dysregulated BCL-6 expression can constitutively repress *PRDM1* (34). The TF Spi-B also represses *PRDM1* expression (171). Spi-B is highly upregulated in ABC-DLBCLs promoting PRDM1 inactivation (172, 173). Experiments using mouse models further suggest that development of ABC DLBCL is a consequence of both BLIMP-1 inactivation and constitutive NF-κB activation (174).

#### 3.1.5 *POU2AF1/*BOB.1/OBF.1

Dysregulated BOB.1/OBF.1 expression in GC-derived lymphomas including FL, BL, DLBCL and HL has been reported by multiple studies. High expression levels of BOB.1/OBF.1 have been found in GC-derived lymphomas (FL, BL, DLBCL), whereas its expression could not be detected in lymphomas that represent other stages of B cell development (175–177). A study by Wang et al. identified BOB.1/OBF.1 as essential for BL cell lines (178). Moreover, others described a requirement of BOB.1/OBF.1 for proliferation and survival of DLBCL cells (179, 180). Chapuy et al. identified a DLBCL-specific super-enhancer near the BOB.1/OBF.1 promoter controlling its expression (179). Inhibition of this super-enhancer impaired DLBCL proliferation (179). Later it was shown that BOB.1/OBF.1 seems to mediate its effects in DLBCL by interaction with Oct-2 (180). Thus, targeting the BOB.1/OBF.1-Oct-2 interaction could be an effective therapeutic strategy in DLBCL (180). Most recently, Song et al. revealed that the fast proliferation of GC-derived lymphoma cells is dependent on BOB.1/OBF.1 (68). Mechanistically, BOB.1/OBF.1 is repressing IRF4 thereby driving GC-derived lymphoma cell proliferation (68). Interestingly, lymphoma cells with reduced BOB.1/OBF.1 expression adopt features associated with favorable prognosis suggesting the transcriptional co-activator a valuable prognostic marker (68).

In contrast, BOB.1/OBF.1 expression is aberrantly silenced in classical Hodgkin lymphoma (cHL). Hodgkin and Reed-Sternberg cells (HRS) are a malignant component of cHL. These cells are characterized by the extinction of B cell program including Ig production (181). The HRS cells lack expression of B cell specific TFs BOB.1/OBF.1 and Oct-2 (182–185). Mechanistically, there is evidence that downregulation of B cell specific genes including BOB.1/OBF.1 in HRS cells is a result of promoter hypermethylation (186). Possibly, the silencing of BOB.1/OBF.1 combined with additional mutations might have a causative role in classical HL.

Besides aberrant expression, BOB.1/OBF.1 chromosomal rearrangements or mutations involved in lymphomagenesis have also been reported. Fusion of LAZ3/BCL-6 and BOB.1/OBF.1 genes by chromosomal translocation have been revealed (187–189). Additionally, a germline variation in the 3′-untranslated region of the *POU2AF1* gene was shown to be associated with susceptibility to lymphoma (190).

In total, all of these studies underline the involvement of BOB.1/OBF.1 in lymphomagenesis, highlighting its diagnostic and therapeutic potential.

Besides lymphomagenesis, deregulation of the transcriptional program regulating the GC reaction can also lead to imbalanced immune responses driving autoimmunity. In particular, deregulated expression of BOB.1/OBF.1 was shown to contribute to the pathogenesis of several autoimmune diseases (191–194). As its contribution to autoimmunity is thoroughly reviewed elsewhere (195), we rather focused on its role in driving lymphomagenesis in this review.

## 4 Conclusion and perspectives

Understanding of the GC biology has made considerable progress in recent years. An effective GC reaction needs interaction of several cell types including reticular cells, FDCs, DCs, GC B, Tfh and Tfr cells. This complex network is coordinated by multiple distinct TFs, which became increasingly clear over the past years. However, it is still not completely resolved how exactly these TFs perform their function. Also, further regulatory factors including co-activators, co-repressors, miRNAs as well as post-transcriptional or epigenetic regulations need to be considered. The requirement of the transcriptional co-activator BOB.1/OBF.1 for the GC reaction not only in B, but also in T cells became more and more evident in recent years. Nevertheless, further studies are required to understand its role especially in Tfr cells. Uncovering mechanisms that have not yet been fully understood, will also help to improve our understanding of dysregulations driving GC-derived lymphomagenesis and therefore help to define new therapeutic approaches to treat GC-derived B malignancies.

## Author contributions

AB wrote the manuscript and prepared visualizations. AU and CB reviewed and edited the manuscript. All authors contributed to the article and approved the submitted version.

## References

[1] De Silva NS Klein U. Dynamics of B cells in germinal centres. Nat Rev Immunol (2015) 15(3):137–48. doi: 10.1038/nri3804 PMC4399774 25656706

[2] Victora GD Nussenzweig MC. Germinal centers. Annu Rev Immunol (2022) 40(1):413–42. doi: 10.1146/annurev-immunol-120419-022408 35113731

[3] Chang JE Turley SJ. Stromal infrastructure of the lymph node and coordination of immunity. Trends Immunol (2015) 36(1):30–9. doi: 10.1016/j.it.2014.11.003 25499856

[4] Turner JS Benet ZL Grigorova IL. Signals 1, 2 and b cell fate or: Where, when and for how long? Immunol Rev (2020) 296(1):9–23. doi: 10.1111/imr.12865 32470215

[5] Phan TG Gray EE Cyster JG. The microanatomy of B cell activation. Curr Opin Immunol (2009) 21(3):258–65. doi: 10.1016/j.coi.2009.05.006 PMC3736860 19481917

[6] Mebius RE Kraal G. Structure and function of the spleen. Nat Rev Immunol (2005) 5(8):606–16. doi: 10.1038/nri1669 16056254

[7] Batista FD Harwood NE. The who, how and where of antigen presentation to B cells. Nat Rev Immunol (2009) 9(1):15–27. doi: 10.1038/nri2454 19079135

[8] McShane AN Malinova D. The ins and outs of antigen uptake in B cells. Front Immunol (2022) 13:892169. doi: 10.3389/fimmu.2022.892169 35572544 PMC9097226

[9] Reif K Ekland EH Ohl L Nakano H Lipp M Förster R. Balanced responsiveness to chemoattractants from adjacent zones determines B-cell position. Nature. (2002) 416(6876):94–9. doi: 10.1038/416094a 11882900

[10] Hardtke S Ohl L Förster R. Balanced expression of CXCR5 and CCR7 on follicular T helper cells determines their transient positioning to lymph node follicles and is essential for efficient B-cell help. Blood. (2005) 106(6):1924–31. doi: 10.1182/blood-2004-11-4494 15899919

[11] Recaldin T Fear DJ. Transcription factors regulating B cell fate in the germinal centre. Clin Exp Immunol (2016) 183(1):65–75. doi: 10.1111/cei.12702 26352785 PMC4687514

[12] Kelly LM Pereira JP Yi T Xu Y Cyster JG. EBI2 guides serial movements of activated B cells and ligand activity is detectable in lymphoid and nonlymphoid tissues. J Immunol (Baltimore Md 1950). (2011) 187(6):3026–32. doi: 10.4049/jimmunol.1101262 PMC3169736 21844396

[13] Suan D Nguyen A Moran I Bourne K Hermes JR Arshi M. T Follicular helper cells have distinct modes of migration and molecular signatures in naive and memory immune responses. Immunity. (2015) 42(4):704–18. doi: 10.1016/j.immuni.2015.03.002 25840682

[14] Kerfoot SM Yaari G Patel JR Johnson KL Gonzalez DG Kleinstein SH. Germinal center B cell and T follicular helper cell development initiates in the interfollicular zone. Immunity. (2011) 34(6):947–60. doi: 10.1016/j.immuni.2011.03.024 PMC3280079 21636295

[15] Stebegg M Kumar SD Silva-Cayetano A Fonseca VR Linterman MA Graca L. Regulation of the germinal center response. Front Immunol (2018) 9:2469. doi: 10.3389/fimmu.2018.02469 30410492 PMC6209676

[16] Pereira JP Kelly LM Cyster JG. Finding the right niche: B-cell migration in the early phases of T-dependent antibody responses. Int Immunol (2010) 22(6):413–9. doi: 10.1093/intimm/dxq047 PMC2877811 20508253

[17] Green JA Cyster JG. S1PR2 links germinal center confinement and growth regulation. Immunol Rev (2012) 247(1):36–51. doi: 10.1111/j.1600-065X.2012.01114.x 22500830 PMC3335345

[18] Moriyama S Takahashi N Green JA Hori S Kubo M Cyster JG. Sphingosine-1-phosphate receptor 2 is critical for follicular helper T cell retention in germinal centers. J Exp Med (2014) 211(7):1297–305. doi: 10.1084/jem.20131666 PMC4076581 24913235

[19] Elsner RA Shlomchik MJ. Germinal center and extrafollicular B cell responses in vaccination, immunity, and autoimmunity. Immunity. (2020) 53(6):1136–50. doi: 10.1016/j.immuni.2020.11.006 PMC7748291 33326765

[20] MacLennan IC Toellner KM Cunningham AF Serre K Sze DM Zúñiga E. Extrafollicular antibody responses. Immunol Rev (2003) 194:8–18. doi: 10.1034/j.1600-065X.2003.00058.x 12846803

[21] Mueller SN Germain RN. Stromal cell contributions to the homeostasis and functionality of the immune system. Nat Rev Immunol (2009) 9(9):618–29. doi: 10.1038/nri2588 PMC2785037 19644499

[22] Laidlaw BJ Cyster JG. Transcriptional regulation of memory B cell differentiation. Nat Rev Immunol (2021) 21(4):209–20. doi: 10.1038/s41577-020-00446-2 PMC7538181 33024284

[23] Song S Matthias PD. The transcriptional regulation of germinal center formation. Front Immunol (2018) 9:2026–. doi: 10.3389/fimmu.2018.02026 PMC6134015 30233601

[24] Ochiai K Maienschein-Cline M Simonetti G Chen J Rosenthal R Brink R. Transcriptional regulation of germinal center B and plasma cell fates by dynamical control of IRF4. Immunity. (2013) 38(5):918–29. doi: 10.1016/j.immuni.2013.04.009 PMC3690549 23684984

[25] Lee CH Melchers M Wang H Torrey TA Slota R Qi CF. Regulation of the germinal center gene program by interferon (IFN) regulatory factor 8/IFN consensus sequence-binding protein. J Exp Med (2006) 203(1):63–72. doi: 10.1084/jem.20051450 16380510 PMC2118063

[26] Ying CY Dominguez-Sola D Fabi M Lorenz IC Hussein S Bansal M. MEF2B mutations lead to deregulated expression of the oncogene BCL6 in diffuse large B cell lymphoma. Nat Immunol (2013) 14(10):1084–92. doi: 10.1038/ni.2688 PMC3954820 23974956

[27] Phan RT Saito M Basso K Niu H Dalla-Favera R. BCL6 interacts with the transcription factor miz-1 to suppress the cyclin-dependent kinase inhibitor p21 and cell cycle arrest in germinal center b cells. Nat Immunol (2005) 6(10):1054–60. doi: 10.1038/ni1245 16142238

[28] Phan RT Dalla-Favera R. The BCL6 proto-oncogene suppresses p53 expression in germinal-centre b cells. Nature. (2004) 432(7017):635–9. doi: 10.1038/nature03147 15577913

[29] Ranuncolo SM Polo JM Dierov J Singer M Kuo T Greally J. Bcl-6 mediates the germinal center B cell phenotype and lymphomagenesis through transcriptional repression of the DNA-damage sensor ATR. Nat Immunol (2007) 8(7):705–14. doi: 10.1038/ni1478 17558410

[30] Ranuncolo SM Polo JM Melnick A. BCL6 represses CHEK1 and suppresses DNA damage pathways in normal and malignant B-cells. Blood cells molecules diseases. (2008) 41(1):95–9. doi: 10.1016/j.bcmd.2008.02.003 PMC2723786 18346918

[31] Basso K Saito M Sumazin P Margolin AA Wang K Lim W-K. Integrated biochemical and computational approach identifies BCL6 direct target genes controlling multiple pathways in normal germinal center B cells. Blood. (2010) 115(5):975–84. doi: 10.1182/blood-2009-06-227017 PMC2817639 19965633

[32] Huang C Gonzalez David G Cote Christine M Jiang Y Hatzi K Teater M. The BCL6 RD2 domain governs commitment of activated B cells to form germinal centers. Cell Rep (2014) 8(5):1497–508. doi: 10.1016/j.celrep.2014.07.059 PMC4163070 25176650

[33] Kitano M Moriyama S Ando Y Hikida M Mori Y Kurosaki T. Bcl6 protein expression shapes pre-germinal center B cell dynamics and follicular helper T cell heterogeneity. Immunity. (2011) 34(6):961–72. doi: 10.1016/j.immuni.2011.03.025 21636294

[34] Tunyaplin C Shaffer AL Angelin-Duclos CD Yu X Staudt LM Calame KL. Direct repression of prdm1 by bcl-6 inhibits plasmacytic differentiation. J Immunol (2004) 173(2):1158–65. doi: 10.4049/jimmunol.173.2.1158 15240705

[35] Muramatsu M Kinoshita K Fagarasan S Yamada S Shinkai Y Honjo T. Class switch recombination and hypermutation require activation-induced cytidine deaminase (AID), a potential RNA editing enzyme. Cell. (2000) 102(5):553–63. doi: 10.1016/S0092-8674(00)00078-7 11007474

[36] Sayegh CE Quong MW Agata Y Murre C. E-proteins directly regulate expression of activation-induced deaminase in mature B cells. Nat Immunol (2003) 4(6):586–93. doi: 10.1038/ni923 12717431

[37] Gonda H Sugai M Nambu Y Katakai T Agata Y Mori KJ. The balance between Pax5 and Id2 activities is the key to AID gene expression. J Exp Med (2003) 198(9):1427–37. doi: 10.1084/jem.20030802 PMC2194241 14581609

[38] Dominguez-Sola D Kung J Holmes AB Wells VA Mo T Basso K. The FOXO1 transcription factor instructs the germinal center dark zone program. Immunity. (2015) 43(6):1064–74. doi: 10.1016/j.immuni.2015.10.015 26620759

[39] Sander S Chu Van T Yasuda T Franklin A Graf R Calado Dinis P. PI3 kinase and FOXO1 transcription factor activity differentially control B cells in the germinal center light and dark zones. Immunity. (2015) 43(6):1075–86. doi: 10.1016/j.immuni.2015.10.021 26620760

[40] Brown AK Webb AE. Regulation of FOXO factors in mammalian cells. Curr topics Dev Biol (2018) 127:165–92. doi: 10.1016/bs.ctdb.2017.10.006 PMC6383790 29433737

[41] Graves DT Milovanova TN. Mucosal immunity and the FOXO1 transcription factors. Front Immunol (2019) 10. doi: 10.3389/fimmu.2019.02530 PMC6896163 31849924

[42] Asada S Daitoku H Matsuzaki H Saito T Sudo T Mukai H. Mitogen-activated protein kinases, erk and p38, phosphorylate and regulate Foxo1. Cell signalling. (2007) 19(3):519–27. doi: 10.1016/j.cellsig.2006.08.015 17113751

[43] Inoue T Shinnakasu R Ise W Kawai C Egawa T Kurosaki T. The transcription factor Foxo1 controls germinal center b cell proliferation in response to T cell help. J Exp Med (2017) 214(4):1181–98. doi: 10.1084/jem.20161263 PMC5379976 28351982

[44] Sciammas R Shaffer AL Schatz JH Zhao H Staudt LM Singh H. Graded expression of interferon regulatory factor-4 coordinates isotype switching with plasma cell differentiation. Immunity. (2006) 25(2):225–36. doi: 10.1016/j.immuni.2006.07.009 16919487

[45] Hauser J Grundström C Kumar R Grundström T. Regulated localization of an AID complex with E2A, PAX5 and IRF4 at the igh locus. Mol Immunol (2016) 80:78–90. doi: 10.1016/j.molimm.2016.10.014 27835756

[46] Ise W Kohyama M Schraml BU Zhang T Schwer B Basu U. The transcription factor BATF controls the global regulators of class-switch recombination in both b cells and T cells. Nat Immunol (2011) 12(6):536–43. doi: 10.1038/ni.2037 PMC3117275 21572431

[47] Kwon H Thierry-Mieg D Thierry-Mieg J Kim H-P Oh J Tunyaplin C. Analysis of interleukin-21-Induced Prdm1 gene regulation reveals functional cooperation of STAT3 and IRF4 transcription factors. Immunity. (2009) 31(6):941–52. doi: 10.1016/j.immuni.2009.10.008 PMC3272079 20064451

[48] Turner CA Jr. Mack DH Davis MM. Blimp-1, a novel zinc finger-containing protein that can drive the maturation of B lymphocytes into immunoglobulin-secreting cells. Cell. (1994) 77(2):297–306. doi: 10.1016/0092-8674(94)90321-2 8168136

[49] Shapiro-Shelef M Lin KI McHeyzer-Williams LJ Liao J McHeyzer-Williams MG Calame K. Blimp-1 is required for the formation of immunoglobulin secreting plasma cells and pre-plasma memory B cells. Immunity. (2003) 19(4):607–20. doi: 10.1016/S1074-7613(03)00267-X 14563324

[50] Minnich M Tagoh H Bönelt P Axelsson E Fischer M Cebolla B. Multifunctional role of the transcription factor blimp-1 in coordinating plasma cell differentiation. Nat Immunol (2016) 17(3):331–43. doi: 10.1038/ni.3349 PMC5790184 26779602

[51] Lin KI Angelin-Duclos C Kuo TC Calame K. Blimp-1-dependent repression of pax-5 is required for differentiation of b cells to immunoglobulin m-secreting plasma cells. Mol Cell Biol (2002) 22(13):4771–80. doi: 10.1128/MCB.22.13.4771-4780.2002 PMC133916 12052884

[52] Reimold AM Iwakoshi NN Manis J Vallabhajosyula P Szomolanyi-Tsuda E Gravallese EM. Plasma cell differentiation requires the transcription factor XBP-1. Nature. (2001) 412(6844):300–7. doi: 10.1038/35085509 11460154

[53] Klein U Casola S Cattoretti G Shen Q Lia M Mo T. Transcription factor IRF4 controls plasma cell differentiation and class-switch recombination. Nat Immunol (2006) 7(7):773–82. doi: 10.1038/ni1357 16767092

[54] Shaffer AL Shapiro-Shelef M Iwakoshi NN Lee AH Qian SB Zhao H. XBP1, downstream of blimp-1, expands the secretory apparatus and other organelles, and increases protein synthesis in plasma cell differentiation. Immunity. (2004) 21(1):81–93. doi: 10.1016/j.immuni.2004.06.010 15345222

[55] Chiu YK Lin IY Su ST Wang KH Yang SY Tsai DY. Transcription factor ABF-1 suppresses plasma cell differentiation but facilitates memory B cell formation. J Immunol (Baltimore Md 1950). (2014) 193(5):2207–17. doi: 10.4049/jimmunol.1400411 25070843

[56] Dominguez-Sola D Victora GD Ying CY Phan RT Saito M Nussenzweig MC. The proto-oncogene MYC is required for selection in the germinal center and cyclic reentry. Nat Immunol (2012) 13(11):1083–91. doi: 10.1038/ni.2428 PMC3711534 23001145

[57] Calado DP Sasaki Y Godinho SA Pellerin A Köchert K Sleckman BP. The cell-cycle regulator c-myc is essential for the formation and maintenance of germinal centers. Nat Immunol (2012) 13(11):1092–100. doi: 10.1038/ni.2418 PMC4132664 23001146

[58] Brunner C Wirth T. BOB.1/OBF.1 - A critical regulator of B cell function. Curr Immunol Rev (2006) 2(1):3–12. doi: 10.2174/157339506775471901

[59] Falkner FG Zachau HG. Correct transcription of an immunoglobulin kappa gene requires an upstream fragment containing conserved sequence elements. Nature. (1984) 310(5972):71–4. doi: 10.1038/310071a0 6330567

[60] Parslow TG Blair DL Murphy WJ Granner DK. Structure of the 5' ends of immunoglobulin genes: A novel conserved sequence. Proc Natl Acad Sci U S A. (1984) 81(9):2650–4. doi: 10.1073/pnas.81.9.2650 PMC345127 6425835

[61] Mueller K Quandt J Marienfeld RB Weihrich P Fiedler K Claussnitzer M. Octamer-dependent transcription in T cells is mediated by NFAT and NF-κB. Nucleic Acids Res (2013) 41(4):2138–54. doi: 10.1093/nar/gks1349 PMC3575799 23293002

[62] Gstaiger M Georgiev O van Leeuwen H van der Vliet P Schaffner W. The b cell coactivator Bob1 shows DNA sequence-dependent complex formation with Oct-1/Oct-2 factors, leading to differential promoter activation. EMBO J (1996) 15(11):2781–90. doi: 10.1002/j.1460-2075.1996.tb00638.x PMC450214 8654375

[63] Luo Y Roeder RG. Cloning, functional characterization, and mechanism of action of the b-cell-specific transcriptional coactivator OCA-b. Mol Cell Biol (1995) 15(8):4115–24. doi: 10.1128/MCB.15.8.4115 PMC230650 7623806

[64] Cepek KL Chasman DI Sharp PA. Sequence-specific DNA binding of the B-cell-specific coactivator OCA-b. Genes Dev (1996) 10(16):2079–88. doi: 10.1101/gad.10.16.2079 8769650

[65] Chasman D Cepek K Sharp PA Pabo CO. Crystal structure of an OCA-B peptide bound to an Oct-1 POU domain/octamer DNA complex: Specific recognition of a protein-DNA interface. Genes Dev (1999) 13(20):2650–7. doi: 10.1101/gad.13.20.2650 PMC317104 10541551

[66] Brunner C Laumen H Nielsen PJ Kraut N Wirth T. Expression of the aldehyde dehydrogenase 2-like gene is controlled by BOB.1/OBF.1 B lymphocytes. J Biol Chem (2003) 278(46):45231–9. doi: 10.1074/jbc.M302539200 12947107

[67] Brunner C Sindrilaru A Girkontaite I Fischer KD Sunderkötter C Wirth T. BOB.1/OBF.1 controls the balance of TH1 and TH2 immune responses. EMBO J (2007) 26(13):3191–202. doi: 10.1038/sj.emboj.7601742 PMC1914090 17568779

[68] Song S Cao C Choukrallah MA Tang F Christofori G Kohler H. OBF1 and Oct factors control the germinal center transcriptional program. Blood. (2021) 137(21):2920–34. doi: 10.1182/blood.2020010175 33512466

[69] Stauss D Brunner C Berberich-Siebelt F Hopken UE Lipp M Muller G. The transcriptional coactivator Bob1 promotes the development of follicular T helper cells *via* Bcl6. EMBO J (2016) 35(8):881–98. doi: 10.15252/embj.201591459 PMC4972135 26957522

[70] Corcoran LM Hasbold J Dietrich W Hawkins E Kallies A Nutt SL. Differential requirement for OBF-1 during antibody-secreting cell differentiation. J Exp Med (2005) 201(9):1385–96. doi: 10.1084/jem.20042325 PMC2213195 15867091

[71] Hess J Nielsen PJ Fischer KD Bujard H Wirth T. The B lymphocyte-specific coactivator BOB.1/OBF.1 is required at multiple stages of b-cell development. Mol Cell Biol (2001) 21(5):1531–9. doi: 10.1128/MCB.21.5.1531-1539.2001 PMC86699 11238890

[72] Schubart DB Rolink A Schubart K Matthias P. Cutting edge: Lack of peripheral B cells and severe agammaglobulinemia in mice simultaneously lacking bruton’s tyrosine kinase and the b cell-specific transcriptional coactivator OBF-1. J Immunol (2000) 164(1):18–22. doi: 10.4049/jimmunol.164.1.18 10604987

[73] Nielsen PJ Georgiev O Lorenz B Schaffner W. B lymphocytes are impaired in mice lacking the transcriptional co-activator Bob1/OCA-B/OBF1. Eur J Immunol (1996) 26(12):3214–8. doi: 10.1002/eji.1830261255 8977324

[74] Samardzic T Marinkovic D Nielsen PJ Nitschke L Wirth T. BOB.1/OBF.1 deficiency affects marginal-zone b-cell compartment. Mol Cell Biol (2002) 22(23):8320–31. doi: 10.1128/MCB.22.23.8320-8331.2002 PMC134056 12417733

[75] Schubart DB Rolink A Kosco-Vilbois MH Botteri F Matthias P. B-cell-specific coactivator OBF-1/OCA-B/Bob1 required for immune response and germinal centre formation. Nature. (1996) 383:538. doi: 10.1038/383538a0 8849727

[76] Betzler AC Fiedler K Hoffmann TK Fehling HJ Wirth T Brunner C. BOB.1/OBF.1 is required during b-cell ontogeny for B-cell differentiation and germinal center function. Eur J Immunol (2022) 52(3):404–17. doi: 10.1002/eji.202149333 34918350

[77] Wolf I Pevzner V Kaiser E Bernhardt G Claudio E Siebenlist U. Downstream activation of a TATA-less promoter by Oct-2, Bob1, and NF-kappaB directs expression of the homing receptor BLR1 to mature B cells. J Biol Chem (1998) 273(44):28831–6. doi: 10.1074/jbc.273.44.28831 9786883

[78] Betzler AC Fiedler K Kokai E Wirth T Hoffmann TK Brunner C. Impaired peyer's patch development in BOB.1/OBF.1-deficient mice. Eur J Immunol (2021) 51(7):1860–3. doi: 10.1002/eji.202048578 33733501

[79] Su GH Chen HM Muthusamy N Garrett-Sinha LA Baunoch D Tenen DG. Defective B cell receptor-mediated responses in mice lacking the ets protein, spi-b. EMBO J (1997) 16(23):7118–29. doi: 10.1093/emboj/16.23.7118 PMC1170313 9384589

[80] Bartholdy B Du Roure C Bordon A Emslie D Corcoran LM Matthias P. The ets factor spi-b is a direct critical target of the coactivator OBF-1. Proc Natl Acad Sci (2006) 103(31):11665–70. doi: 10.1073/pnas.0509430103 PMC1513538 16861304

[81] Chu CS Hellmuth JC Singh R Ying HY Skrabanek L Teater MR. Unique immune cell coactivators specify locus control region function and cell stage. Mol Cell (2020) 80(5):845–61.e10. doi: 10.1016/j.molcel.2020.10.036 33232656 PMC7737631

[82] Choi Youn S Kageyama R Eto D Escobar Tania C Johnston Robert J Monticelli L. ICOS receptor instructs T follicular helper cell versus effector cell differentiation *via* induction of the transcriptional repressor Bcl6. Immunity. (2011) 34(6):932–46. doi: 10.1016/j.immuni.2011.03.023 PMC3124577 21636296

[83] DiToro D Winstead CJ Pham D Witte S Andargachew R Singer JR. Differential IL-2 expression defines developmental fates of follicular versus nonfollicular helper T cells. Science (2018) 361(6407):eaao2933. doi: 10.1126/science.aao2933 30213884 PMC6501592

[84] Nurieva RI Chung Y Hwang D Yang XO Kang HS Ma L. Generation of T follicular helper cells is mediated by interleukin-21 but independent of T helper 1, 2, or 17 cell lineages. Immunity. (2008) 29(1):138–49. doi: 10.1016/j.immuni.2008.05.009 PMC2556461 18599325

[85] Choi YS Eto D Yang JA Lao C Crotty S. Cutting edge: STAT1 is required for IL-6-mediated Bcl6 induction for early follicular helper cell differentiation. J Immunol (Baltimore Md 1950). (2013) 190(7):3049–53. doi: 10.4049/jimmunol.1203032 PMC3626564 23447690

[86] Vinuesa CG Linterman MA Yu D MacLennan IC. Follicular helper T cells. Annu Rev Immunol (2016) 34:335–68. doi: 10.1146/annurev-immunol-041015-055605 26907215

[87] Nakayamada S Poholek AC Lu KT Takahashi H Kato M Iwata S. Type I IFN induces binding of STAT1 to Bcl6: Divergent roles of STAT family transcription factors in the T follicular helper cell genetic program. J Immunol (Baltimore Md 1950). (2014) 192(5):2156–66. doi: 10.4049/jimmunol.1300675 PMC3967131 24489092

[88] Choi YS Gullicksrud JA Xing S Zeng Z Shan Q Li F. LEF-1 and TCF-1 orchestrate TFH differentiation by regulating differentiation circuits upstream of the transcriptional repressor Bcl6. Nat Immunol (2015) 16(9):980–90. doi: 10.1038/ni.3226 PMC4545301 26214741

[89] Xu L Cao Y Xie Z Huang Q Bai Q Yang X. The transcription factor TCF-1 initiates the differentiation of TFH cells during acute viral infection. Nat Immunol (2015) 16(9):991–9. doi: 10.1038/ni.3229 26214740

[90] Ribeiro F Perucha E Graca L. T Follicular cells: The regulators of germinal center homeostasis. Immunol Lett (2022) 244:1–11. doi: 10.1016/j.imlet.2022.02.008 35227735

[91] Yu D Rao S Tsai LM Lee SK He Y Sutcliffe EL. The transcriptional repressor bcl-6 directs T follicular helper cell lineage commitment. Immunity. (2009) 31(3):457–68. doi: 10.1016/j.immuni.2009.07.002 19631565

[92] Liu X Chen X Zhong B Wang A Wang X Chu F. Transcription factor achaete-scute homologue 2 initiates follicular T-helper-cell development. Nature. (2014) 507(7493):513–8. doi: 10.1038/nature12910 PMC4012617 24463518

[93] Weber JP Fuhrmann F Feist RK Lahmann A Al Baz MS Gentz LJ. ICOS maintains the T follicular helper cell phenotype by down-regulating krüppel-like factor 2. J Exp Med (2015) 212(2):217–33. doi: 10.1084/jem.20141432 PMC4322049 25646266

[94] Hiramatsu Y Suto A Kashiwakuma D Kanari H S-i K Ikeda K. C-maf activates the promoter and enhancer of the IL-21 gene, and TGF-β inhibits c-maf-induced IL-21 production in CD4+ T cells. J leukocyte Biol (2010) 87(4):703–12. doi: 10.1189/jlb.0909639 20042469

[95] Cannons JL Qi H Lu KT Dutta M Gomez-Rodriguez J Cheng J. Optimal germinal center responses require a multistage T cell: B cell adhesion process involving integrins, SLAM-associated protein, and CD84. Immunity. (2010) 32(2):253–65. doi: 10.1016/j.immuni.2010.01.010 PMC2830297 20153220

[96] Crotty S. Follicular helper CD4 T cells (TFH). Annu Rev Immunol (2011) 29:621–63. doi: 10.1146/annurev-immunol-031210-101400 21314428

[97] Crotty S. T Follicular helper cell differentiation, function, and roles in disease. Immunity. (2014) 41(4):529–42. doi: 10.1016/j.immuni.2014.10.004 PMC4223692 25367570

[98] Shulman Z Gitlin AD Targ S Jankovic M Pasqual G Nussenzweig MC. T Follicular helper cell dynamics in germinal centers. Science. (2013) 341(6146):673–7. doi: 10.1126/science.1241680 PMC3941467 23887872

[99] Yamashita K Kawata K Matsumiya H Kamekura R Jitsukawa S Nagaya T. Bob1 limits cellular frequency of T-follicular helper cells. Eur J Immunol (2016) 46(6):1361–70. doi: 10.1002/eji.201545499 PMC5084739 27080143

[100] Chtanova T Tangye SG Newton R Frank N Hodge MR Rolph MS. T Follicular helper cells express a distinctive transcriptional profile, reflecting their role as non-Th1/Th2 effector cells that provide help for B cells. J Immunol (2004) 173(1):68–78. doi: 10.4049/jimmunol.173.1.68 15210760

[101] Locci M Havenar-Daughton C Landais E Wu J Kroenke Mark A Arlehamn Cecilia L. Human circulating PD-1+CXCR3-CXCR5+ memory tfh cells are highly functional and correlate with broadly neutralizing HIV antibody responses. Immunity. (2013) 39(4):758–69. doi: 10.1016/j.immuni.2013.08.031 PMC3996844 24035365

[102] Karnowski A Chevrier S Belz GT Mount A Emslie D D'Costa K. B and T cells collaborate in antiviral responses *via* IL-6, IL-21, and transcriptional activator and coactivator, Oct2 and OBF-1. J Exp Med (2012) 209(11):2049–64. doi: 10.1084/jem.20111504 PMC3478936 23045607

[103] Kury P Staniek J Wegehaupt O Janowska I Eckenweiler M Korinthenberg R. Agammaglobulinemia with normal B-cell numbers in a patient lacking Bob1. J Allergy Clin Immunol (2021) 147(5):1977–80. doi: 10.1016/j.jaci.2021.01.027 33571536

[104] Lombard-Vadnais F Lacombe J Chabot-Roy G Ferron M Lesage S. OCA-b does not act as a transcriptional coactivator in T cells. Immunol Cell Biol (2022) 100(5):338–51. doi: 10.1111/imcb.12543 35285071

[105] Betzler AC Ezić J Abou Kors T Hoffmann TK Wirth T Brunner C. T Cell specific BOB.1/OBF.1 expression promotes germinal center response and T helper cell differentiation. Front Immunol (2022) 13:889564. doi: 10.3389/fimmu.2022.889564 35603192 PMC9114770

[106] Chevrier S Kratina T Emslie D Karnowski A Corcoran LM. Germinal center-independent, IgM-mediated autoimmunity in sanroque mice lacking Obf1. Immunol Cell Biol (2014) 92(1):12–9. doi: 10.1038/icb.2013.71 24217807

[107] Chung Y Tanaka S Chu F Nurieva RI Martinez GJ Rawal S. Follicular regulatory T cells expressing Foxp3 and bcl-6 suppress germinal center reactions. Nat Med (2011) 17(8):983–8. doi: 10.1038/nm.2426 PMC3151340 21785430

[108] Linterman MA Pierson W Lee SK Kallies A Kawamoto S Rayner TF. Foxp3+ follicular regulatory T cells control the germinal center response. Nat Med (2011) 17(8):975–82. doi: 10.1038/nm.2425 PMC3182542 21785433

[109] Wollenberg I Agua-Doce A Hernández A Almeida C Oliveira VG Faro J. Regulation of the germinal center reaction by Foxp3+ follicular regulatory T cells. J Immunol (Baltimore Md 1950). (2011) 187(9):4553–60. doi: 10.4049/jimmunol.1101328 21984700

[110] Amé-Thomas P Le Priol J Yssel H Caron G Pangault C Jean R. Characterization of intratumoral follicular helper T cells in follicular lymphoma: Role in the survival of malignant B cells. Leukemia. (2012) 26(5):1053–63. doi: 10.1038/leu.2011.301 PMC3428269 22015774

[111] Wing JB Kitagawa Y Locci M Hume H Tay C Morita T. A distinct subpopulation of CD25(-) T-follicular regulatory cells localizes in the germinal centers. Proc Natl Acad Sci U S A (2017) 114(31):E6400–e9. doi: 10.1073/pnas.1705551114 PMC5547636 28698369

[112] Fonseca VR Agua-Doce A Maceiras AR Pierson W Ribeiro F Romão VC. Human blood t(fr) cells are indicators of ongoing humoral activity not fully licensed with suppressive function. Sci Immunol (2017) 2(14). doi: 10.1126/sciimmunol.aan1487 PMC7116402 28802258

[113] Xie MM Dent AL. Unexpected help: Follicular regulatory T cells in the germinal center. Front Immunol (2018) 9. doi: 10.3389/fimmu.2018.01536 PMC6036241 30013575

[114] Jacobsen JT Hu W BRC T Solem S Galante A Lin Z. Expression of Foxp3 by T follicular helper cells in end-stage germinal centers. Science (2021) 373(6552). doi: 10.1126/science.abe5146 PMC9007630 34437125

[115] Hao H Nakayamada S Tanaka Y. Differentiation, functions, and roles of T follicular regulatory cells in autoimmune diseases. Inflammation Regeneration. (2021) 41(1):14. doi: 10.1186/s41232-021-00164-9 33934711 PMC8088831

[116] Sage PT Paterson AM Lovitch SB Sharpe AH. The coinhibitory receptor CTLA-4 controls B cell responses by modulating T follicular helper, T follicular regulatory, and T regulatory cells. Immunity. (2014) 41(6):1026–39. doi: 10.1016/j.immuni.2014.12.005 PMC4309019 25526313

[117] Fahlquist Hagert C Degn SE. T Follicular regulatory cells: Guardians of the germinal centre? Scandinavian J Immunol (2020) 92(4):e12942. doi: 10.1111/sji.12942 PMC7583367 32697349

[118] Aloulou M Carr EJ Gador M Bignon A Liblau RS Fazilleau N. Follicular regulatory T cells can be specific for the immunizing antigen and derive from naive T cells. Nat Commun (2016) 7:10579. doi: 10.1038/ncomms10579 26818004 PMC4738360

[119] Ritvo PG Churlaud G Quiniou V Florez L Brimaud F Fourcade G. T(fr) cells lack IL-2Rα but express decoy IL-1R2 and IL-1Ra and suppress the IL-1-dependent activation of t(fh) cells. Sci Immunol (2017) 2(15). doi: 10.1126/sciimmunol.aan0368 28887367

[120] Zhang R Sage PT Finn K Huynh A Blazar BR Marangoni F. B cells drive autoimmunity in mice with CD28-deficient regulatory T cells. J Immunol (Baltimore Md 1950). (2017) 199(12):3972–80. doi: 10.4049/jimmunol.1700409 PMC5716898 29093061

[121] Maceiras AR Almeida SCP Mariotti-Ferrandiz E Chaara W Jebbawi F Six A. T Follicular helper and T follicular regulatory cells have different TCR specificity. Nat Commun (2017) 8:15067. doi: 10.1038/ncomms15067 28429709 PMC5413949

[122] Yang G Yang X Zhang J Li G Zheng D Peng A. Transcriptional repressor Blimp1 regulates follicular regulatory T-cell homeostasis and function. Immunology. (2018) 153(1):105–17. doi: 10.1111/imm.12815 PMC5721240 28833081

[123] Hou S Clement RL Diallo A Blazar BR Rudensky AY Sharpe AH. FoxP3 and Ezh2 regulate tfr cell suppressive function and transcriptional program. J Exp Med (2019) 216(3):605–20. doi: 10.1084/jem.20181134 PMC6400538 30705058

[124] Vaeth M Müller G Stauss D Dietz L Klein-Hessling S Serfling E. Follicular regulatory T cells control humoral autoimmunity *via* NFAT2-regulated CXCR5 expression. J Exp Med (2014) 211(3):545–61. doi: 10.1084/jem.20130604 PMC3949566 24590764

[125] Vaeth M Eckstein M Shaw PJ Kozhaya L Yang J Berberich-Siebelt F. Store-operated Ca2+ entry in follicular T cells controls humoral immune responses and autoimmunity. Immunity. (2016) 44(6):1350–64. doi: 10.1016/j.immuni.2016.04.013 PMC4917422 27261277

[126] Leavenworth JW Verbinnen B Yin J Huang H Cantor H. A p85α-osteopontin axis couples the receptor ICOS to sustained bcl-6 expression by follicular helper and regulatory T cells. Nat Immunol (2015) 16(1):96–106. doi: 10.1038/ni.3050 25436971 PMC4405167

[127] Xu L Huang Q Wang H Hao Y Bai Q Hu J. The kinase mTORC1 promotes the generation and suppressive function of follicular regulatory T cells. Immunity. (2017) 47(3):538–51.e5. doi: 10.1016/j.immuni.2017.08.011 28930662

[128] Miyazaki M Miyazaki K Chen S Itoi M Miller M Lu L-F. Id2 and Id3 maintain the regulatory T cell pool to suppress inflammatory disease. Nat Immunol (2014) 15(8):767–76. doi: 10.1038/ni.2928 PMC4365819 24973820

[129] Wu X Wang Y Huang R Gai Q Liu H Shi M. SOSTDC1-producing follicular helper T cells promote regulatory follicular T cell differentiation. Science. (2020) 369(6506):984–8. doi: 10.1126/science.aba6652 32820125

[130] Wheaton JD Yeh CH Ciofani M. Cutting edge: c-maf is required for regulatory T cells to adopt RORγt(+) and follicular phenotypes. J Immunol (Baltimore Md 1950). (2017) 199(12):3931–6. doi: 10.4049/jimmunol.1701134 PMC5728164 29127150

[131] Marson A Kretschmer K Frampton GM Jacobsen ES Polansky JK MacIsaac KD. Foxp3 occupancy and regulation of key target genes during T-cell stimulation. Nature. (2007) 445(7130):931–5. doi: 10.1038/nature05478 PMC3008159 17237765

[132] Metenou S Coulibaly YI Sturdevant D Dolo H Diallo AA Soumaoro L. Highly heterogeneous, activated, and short-lived regulatory T cells during chronic filarial infection. Eur J Immunol (2014) 44(7):2036–47. doi: 10.1002/eji.201444452 PMC4809647 24737144

[133] Sukiennicki TL Fowell DJ. Distinct molecular program imposed on CD4+ T cell targets by CD4+CD25+ regulatory T cells. J Immunol (Baltimore Md 1950). (2006) 177(10):6952–61. doi: 10.4049/jimmunol.177.10.6952 17082610

[134] Sage PT Ron-Harel N Juneja VR Sen DR Maleri S Sungnak W. Suppression by T(FR) cells leads to durable and selective inhibition of B cell effector function. Nat Immunol (2016) 17(12):1436–46. doi: 10.1038/ni.3578 PMC5502675 27695002

[135] Placek K Hu G Cui K Zhang D Ding Y Lee JE. MLL4 prepares the enhancer landscape for Foxp3 induction *via* chromatin looping. Nat Immunol (2017) 18(9):1035–45. doi: 10.1038/ni.3812 PMC5836551 28759003

[136] Basso K Dalla-Favera R. Germinal centres and b cell lymphomagenesis. Nat Rev Immunol (2015) 15(3):172–84. doi: 10.1038/nri3814 25712152

[137] Mlynarczyk C Fontán L Melnick A. Germinal center-derived lymphomas: The darkest side of humoral immunity. Immunol Rev (2019) 288(1):214–39. doi: 10.1111/imr.12755 PMC6518944 30874354

[138] Shaffer AL 3rd Young RM Staudt LM. Pathogenesis of human B cell lymphomas. Annu Rev Immunol (2012) 30:565–610. doi: 10.1146/annurev-immunol-020711-075027 22224767 PMC7478144

[139] Küppers R. Mechanisms of b-cell lymphoma pathogenesis. Nat Rev Cancer. (2005) 5(4):251–62. doi: 10.1038/nrc1589 15803153

[140] Nussenzweig A Nussenzweig MC. Origin of chromosomal translocations in lymphoid cancer. Cell. (2010) 141(1):27–38. doi: 10.1016/j.cell.2010.03.016 20371343 PMC2874895

[141] Dalla-Favera R Bregni M Erikson J Patterson D Gallo RC Croce CM. Human c-myc onc gene is located on the region of chromosome 8 that is translocated in burkitt lymphoma cells. Proc Natl Acad Sci U S A (1982) 79(24):7824–7. doi: 10.1073/pnas.79.24.7824 PMC347441 6961453

[142] Taub R Kirsch I Morton C Lenoir G Swan D Tronick S. Translocation of the c-myc gene into the immunoglobulin heavy chain locus in human burkitt lymphoma and murine plasmacytoma cells. Proc Natl Acad Sci U S A (1982) 79(24):7837–41. doi: 10.1073/pnas.79.24.7837 PMC347444 6818551

[143] Schmitz R Ceribelli M Pittaluga S Wright G Staudt LM. Oncogenic mechanisms in burkitt lymphoma. Cold Spring Harbor Perspect Med (2014) 4(2). doi: 10.1101/cshperspect.a014282 PMC3904095 24492847

[144] Li S Lin P Young KH Kanagal-Shamanna R Yin CC Medeiros LJ. MYC/BCL2 double-hit high-grade B-cell lymphoma. Adv anatomic pathology. (2013) 20(5):315–26. doi: 10.1097/PAP.0b013e3182a289f2 23939148

[145] Dominguez-Sola D Ying CY Grandori C Ruggiero L Chen B Li M. Non-transcriptional control of DNA replication by c-myc. Nature. (2007) 448(7152):445–51. doi: 10.1038/nature05953 17597761

[146] Basso K Dalla-Favera R. BCL6: master regulator of the germinal center reaction and key oncogene in B cell lymphomagenesis. Adv Immunol (2010) 105:193–210. doi: 10.1016/S0065-2776(10)05007-8 20510734

[147] Ye BH Chaganti S Chang CC Niu H Corradini P Chaganti RS. Chromosomal translocations cause deregulated BCL6 expression by promoter substitution in B cell lymphoma. EMBO J (1995) 14(24):6209–17. doi: 10.1002/j.1460-2075.1995.tb00311.x PMC394745 8557040

[148] Chen W Iida S Louie DC Dalla-Favera R Chaganti RS. Heterologous promoters fused to BCL6 by chromosomal translocations affecting band 3q27 cause its deregulated expression during B-cell differentiation. Blood. (1998) 91(2):603–7. doi: 10.1182/blood.V91.2.603 9427715

[149] Ichinohasama R Miura I Funato T Sato I Suzuki C Saito Y. A recurrent nonrandom translocation (3;7)(q27;p12) associated with BCL-6 gene rearrangement in B-cell diffuse large cell lymphoma. Cancer Genet cytogenetics. (1998) 104(1):19–27. doi: 10.1016/S0165-4608(97)00412-3 9648553

[150] Pasqualucci L Migliazza A Basso K Houldsworth J Chaganti RS Dalla-Favera R. Mutations of the BCL6 proto-oncogene disrupt its negative autoregulation in diffuse large B-cell lymphoma. Blood. (2003) 101(8):2914–23. doi: 10.1182/blood-2002-11-3387 12515714

[151] Wang X Li Z Naganuma A Ye BH. Negative autoregulation of BCL-6 is bypassed by genetic alterations in diffuse large B cell lymphomas. Proc Natl Acad Sci U S A (2002) 99(23):15018–23. doi: 10.1073/pnas.232581199 PMC137537 12407182

[152] Saito M Gao J Basso K Kitagawa Y Smith PM Bhagat G. A signaling pathway mediating downregulation of BCL6 in germinal center B cells is blocked by BCL6 gene alterations in B cell lymphoma. Cancer Cell (2007) 12(3):280–92. doi: 10.1016/j.ccr.2007.08.011 17785208

[153] Brescia P Schneider C Holmes AB Shen Q Hussein S Pasqualucci L. MEF2B instructs germinal center development and acts as an oncogene in B cell lymphomagenesis. Cancer Cell (2018) 34(3):453–65.e9. doi: 10.1016/j.ccell.2018.08.006 30205047 PMC6223119

[154] Coomans de Brachène A Demoulin J-B. FOXO transcription factors in cancer development and therapy. Cell Mol Life Sci (2016) 73(6):1159–72. doi: 10.1007/s00018-015-2112-y PMC11108379 26686861

[155] Hornsveld M Smits LMM Meerlo M van Amersfoort M Groot Koerkamp MJA van Leenen D. FOXO transcription factors both suppress and support breast cancer progression. Cancer Res (2018) 78(9):2356–69. doi: 10.1158/0008-5472.CAN-17-2511 29440168

[156] Sykes Stephen M Lane Steven W Bullinger L Kalaitzidis D Yusuf R Saez B. AKT/FOXO signaling enforces reversible differentiation blockade in myeloid leukemias. Cell. (2011) 146(5):697–708. doi: 10.1016/j.cell.2011.07.032 21884932 PMC3826540

[157] Kabrani E Chu VT Tasouri E Sommermann T Baßler K Ulas T. Nuclear FOXO1 promotes lymphomagenesis in germinal center B cells. Blood. (2018) 132(25):2670–83. doi: 10.1182/blood-2018-06-856203 30333121

[158] Grande BM Gerhard DS Jiang A Griner NB Abramson JS Alexander TB. Genome-wide discovery of somatic coding and noncoding mutations in pediatric endemic and sporadic burkitt lymphoma. Blood. (2019) 133(12):1313–24. doi: 10.1182/blood-2018-09-871418 PMC6428665 30617194

[159] Pasqualucci L Khiabanian H Fangazio M Vasishtha M Messina M Holmes AB. Genetics of follicular lymphoma transformation. Cell Rep (2014) 6(1):130–40. doi: 10.1016/j.celrep.2013.12.027 PMC4100800 24388756

[160] Morin RD Assouline S Alcaide M Mohajeri A Johnston RL Chong L. Genetic landscapes of relapsed and refractory diffuse Large B-cell lymphomas. Clin Cancer Res an Off J Am Assoc Cancer Res (2016) 22(9):2290–300. doi: 10.1158/1078-0432.CCR-15-2123 26647218

[161] Zhou P Blain AE Newman AM Zaka M Chagaluka G Adlar FR. Sporadic and endemic burkitt lymphoma have frequent FOXO1 mutations but distinct hotspots in the AKT recognition motif. Blood advances. (2019) 3(14):2118–27. doi: 10.1182/bloodadvances.2018029546 PMC6650741 31300419

[162] Trinh DL Scott DW Morin RD Mendez-Lago M An J Jones SJ. Analysis of FOXO1 mutations in diffuse large B-cell lymphoma. Blood. (2013) 121(18):3666–74. doi: 10.1182/blood-2013-01-479865 PMC3643765 23460611

[163] Sablon A Bollaert E Pirson C Velghe AI Demoulin J-B. FOXO1 forkhead domain mutants in B-cell lymphoma lack transcriptional activity. Sci Rep (2022) 12(1):1309. doi: 10.1038/s41598-022-05334-4 35079069 PMC8789783

[164] Brunet A Bonni A Zigmond MJ Lin MZ Juo P Hu LS. Akt promotes cell survival by phosphorylating and inhibiting a forkhead transcription factor. Cell. (1999) 96(6):857–68. doi: 10.1016/S0092-8674(00)80595-4 10102273

[165] Gehringer F Weissinger SE Swier LJ Moller P Wirth T Ushmorov A. FOXO1 confers maintenance of the dark zone proliferation and survival program and can be pharmacologically targeted in burkitt lymphoma. Cancers (Basel). (2019) 11(10):1427. doi: 10.3390/cancers11101427 31557894 PMC6826697

[166] Gehringer F Weissinger SE Moller P Wirth T Ushmorov A. Physiological levels of the PTEN-PI3K-AKT axis activity are required for maintenance of burkitt lymphoma. Leukemia. (2020) 34(3):857–71. doi: 10.1038/s41375-019-0628-0 PMC7214272 31719683

[167] Roberto MP Varano G Vinas-Castells R Holmes AB Kumar R Pasqualucci L. Mutations in the transcription factor FOXO1 mimic positive selection signals to promote germinal center B cell expansion and lymphomagenesis. Immunity. (2021) 54(8):1807–24.e14. doi: 10.1016/j.immuni.2021.07.009 34380064 PMC8475267

[168] Mandelbaum J Bhagat G Tang H Mo T Brahmachary M Shen Q. BLIMP1 is a tumor suppressor gene frequently disrupted in activated B cell-like diffuse large b cell lymphoma. Cancer Cell (2010) 18(6):568–79. doi: 10.1016/j.ccr.2010.10.030 PMC3030476 21156281

[169] Pasqualucci L Compagno M Houldsworth J Monti S Grunn A Nandula SV. Inactivation of the PRDM1/BLIMP1 gene in diffuse large B cell lymphoma. J Exp Med (2006) 203(2):311–7. doi: 10.1084/jem.20052204 PMC2118216 16492805

[170] Tam W Gomez M Chadburn A Lee JW Chan WC Knowles DM. Mutational analysis of PRDM1 indicates a tumor-suppressor role in diffuse large B-cell lymphomas. Blood. (2006) 107(10):4090–100. doi: 10.1182/blood-2005-09-3778 16424392

[171] Schmidlin H Diehl SA Nagasawa M Scheeren FA Schotte R Uittenbogaart CH. Spi-B inhibits human plasma cell differentiation by repressing BLIMP1 and XBP-1 expression. Blood. (2008) 112(5):1804–12. doi: 10.1182/blood-2008-01-136440 PMC2518887 18552212

[172] Lenz G Nagel I Siebert R Roschke AV Sanger W Wright GW. Aberrant immunoglobulin class switch recombination and switch translocations in activated B cell-like diffuse large b cell lymphoma. J Exp Med (2007) 204(3):633–43. doi: 10.1084/jem.20062041 PMC2137913 17353367

[173] Lenz G Wright GW Emre NC Kohlhammer H Dave SS Davis RE. Molecular subtypes of diffuse large B-cell lymphoma arise by distinct genetic pathways. Proc Natl Acad Sci U S A. (2008) 105(36):13520–5. doi: 10.1073/pnas.0804295105 PMC2533222 18765795

[174] Calado DP Zhang B Srinivasan L Sasaki Y Seagal J Unitt C. Constitutive canonical NF-κB activation cooperates with disruption of BLIMP1 in the pathogenesis of activated B cell-like diffuse large cell lymphoma. Cancer Cell (2010) 18(6):580–9. doi: 10.1016/j.ccr.2010.11.024 PMC3018685 21156282

[175] Greiner A Muller KB Hess J Pfeffer K Muller-Hermelink HK Wirth T. Up-regulation of BOB.1/OBF.1 expression in normal germinal center B cells and germinal center-derived lymphomas. Am J Pathol (2000) 156(2):501–7. doi: 10.1016/S0002-9440(10)64754-2 PMC1850056 10666379

[176] Loddenkemper C Anagnostopoulos I Hummel M Jöhrens-Leder K Foss H-D Jundt F. Differential eµ enhancer activity and expression of BOB.1/OBF.1, Oct2, PU.1, and immunoglobulin in reactive B-cell populations, B-cell non-Hodgkin lymphomas, and Hodgkin lymphomas. J Pathol (2004) 202(1):60–9. doi: 10.1002/path.1485 14694522

[177] Sáez A-I Artiga M-J Sánchez-Beato M Sánchez-Verde L García J-F Camacho F-I. Analysis of octamer-binding transcription factors Oct2 and Oct1 and their coactivator BOB.1/OBF.1 in lymphomas. Modern Pathol (2002) 15(3):211–20. doi: 10.1038/modpathol.3880518 11904338

[178] Wang T Birsoy K Hughes NW Krupczak KM Post Y Wei JJ. Identification and characterization of essential genes in the human genome. Science. (2015) 350(6264):1096–101. doi: 10.1126/science.aac7041 PMC4662922 26472758

[179] Chapuy B McKeown MR Lin CY Monti S Roemer MG Qi J. Discovery and characterization of super-enhancer-associated dependencies in diffuse large B cell lymphoma. Cancer Cell (2013) 24(6):777–90. doi: 10.1016/j.ccr.2013.11.003 PMC4018722 24332044

[180] Hodson DJ Shaffer AL Xiao W Wright GW Schmitz R Phelan JD. Regulation of normal b-cell differentiation and malignant B-cell survival by OCT2. Proc Natl Acad Sci (2016) 113(14):E2039–E46. doi: 10.1073/pnas.1600557113 PMC4833274 26993806

[181] Küppers R Klein U Schwering I Distler V Bräuninger A Cattoretti G. Identification of Hodgkin and reed-sternberg cell-specific genes by gene expression profiling. J Clin Invest. (2003) 111(4):529–37. doi: 10.1172/JCI200316624 PMC151925 12588891

[182] Re D Müschen M Ahmadi T Wickenhauser C Staratschek-Jox A Holtick U. Oct-2 and bob-1 deficiency in Hodgkin and reed sternberg cells. Cancer Res (2001) 61(5):2080–4. 11280769

[183] Stein H Marafioti T Foss H-D Laumen H Hummel M Anagnostopoulos I. Down-regulation of BOB.1/OBF.1 and Oct2 in classical Hodgkin disease but not in lymphocyte predominant Hodgkin disease correlates with immunoglobulin transcription. Blood (2001) 97(2):496–501. doi: 10.1182/blood.v97.2.496 11154228

[184] Theil J Laumen H Marafioti T Hummel M Lenz G Wirth T. Defective octamer-dependent transcription is responsible for silenced immunoglobulin transcription in reed-sternberg cells. Blood. (2001) 97(10):3191–6. doi: 10.1182/blood.V97.10.3191 11342448

[185] Hertel CB Zhou XG Hamilton-Dutoit SJ Junker S. Loss of b cell identity correlates with loss of B cell-specific transcription factors in Hodgkin/Reed-sternberg cells of classical Hodgkin lymphoma. Oncogene. (2002) 21(32):4908–20. doi: 10.1038/sj.onc.1205629 12118370

[186] Ushmorov A Leithäuser F Sakk O Weinhäsel A Popov SW Möller P. Epigenetic processes play a major role in B-cell-specific gene silencing in classical Hodgkin lymphoma. Blood. (2006) 107(6):2493–500. doi: 10.1182/blood-2005-09-3765 16304050

[187] Galiègue Zouitina S Quief S Hildebrand MP Denis C Lecocq G Collyn-d'Hooghe M. The b cell transcriptional coactivator BOB1/OBF1 gene fuses to the LAZ3/BCL6 gene by t(3;11)(q27;q23.1) chromosomal translocation in a B cell leukemia line (Karpas 231). Leukemia (1996) 10(4):579–87. 8618432

[188] Galiègue-Zouitina S Quief S Hildebrand MP Denis C Lecocq G Collyn-d'Hooghe M. Fusion of the LAZ3/BCL6 and BOB1/OBF1 genes by t(3; 11) (q27; q23) chromosomal translocation. C R Acad Sci III (1995) 318(11):1125–31. 8574789

[189] Yuille MA Galiegue-Zouitina S Hiorns LR Jadayel D De Schouwer PJ Catovsky D. Heterogeneity of breakpoints at the transcriptional co-activator gene, BOB-1, in lymphoproliferative disease. Leukemia. (1996) 10(9):1492–6. 8751468

[190] Zhai K Chang J Hu J Wu C Lin D. Germline variation in the 3'-untranslated region of the POU2AF1 gene is associated with susceptibility to lymphoma. Mol carcinogenesis. (2017) 56(8):1945–52. doi: 10.1002/mc.22652 28345816

[191] Ikegami I Takaki H Kamiya S Kamekura R Ichimiya S. Bob1 enhances RORγt-mediated IL-17A expression in Th17 cells through interaction with RORγt. Biochem Biophys Res Commun (2019) 514(4):1167–71. doi: 10.1016/j.bbrc.2019.05.057 31103264

[192] Levels MJ Van Tok MN Cantaert T Cañete JD Kroese FG Germar K. The transcriptional coactivator Bob1 is associated with pathologic B cell responses in autoimmune tissue inflammation. Arthritis Rheumatol (Hoboken NJ). (2017) 69(4):750–62. doi: 10.1002/art.39993 27907250

[193] Kim U Qin XF Gong S Stevens S Luo Y Nussenzweig M. The B-cell-specific transcription coactivator OCA-B/OBF-1/Bob-1 is essential for normal production of immunoglobulin isotypes. Nature. (1996) 383(6600):542–7. doi: 10.1038/383542a0 8849728

[194] Zuo J Ge H Zhu G Matthias P Sun J. OBF-1 is essential for the generation of antibody-secreting cells and the development of autoimmunity in MRL-lpr mice. J Autoimmun (2007) 29(2-3):87–96. doi: 10.1016/j.jaut.2007.05.001 17574818

[195] Yeremenko N Danger R Baeten D Tomilin A Brouard S. Transcriptional regulator BOB.1: Molecular mechanisms and emerging role in chronic inflammation and autoimmunity. Autoimmun Rev (2021) 20(6):102833. doi: 10.1016/j.autrev.2021.102833 33864944

